# Association of prenatal exposure to opioids, cannabis, and polysubstance use with cord blood DNA methylation patterns in a multiancestry cohort

**DOI:** 10.1080/15592294.2026.2670885

**Published:** 2026-08-26

**Authors:** Henri Garrison-Desany, Ellen Howerton, Xiumei Hong, Brion Maher, Colleen Pearson, Barry Zuckerman, Guoying Wang, M. Daniele Fallin, Terri Beaty, Liming Liang, Xiaobin Wang, Christine Ladd-Acosta

**Affiliations:** a Department of Epidemiology, Johns Hopkins Bloomberg School of Public Health, Baltimore, MD, USA; b Center on the Early Life Origins of Disease, Department of Population, Family and Reproductive Health, Johns Hopkins Bloomberg School of Public Health, Baltimore, MD, USA; c Department of Mental Health, Johns Hopkins Bloomberg School of Public Health, Baltimore, MD, USA; d Department of Pediatrics, Boston University School of Medicine and Boston Medical Center, Boston, MA, USA; e Rollins School of Public Health, Emory University, Atlanta, GA, USA; f Department of Biostatistics, Harvard T.H. Chan School of Public Health, Boston, MA, USA; g Department of Epidemiology, Harvard T.H. Chan School of Public Health, Boston, MA, USA; h Department of Pediatrics, Johns Hopkins School of Medicine, Baltimore, MD, USA

**Keywords:** Cord blood, cannabis use, epigenome-wide association study, opioid use, prenatal exposure, polysubstance use

## Abstract

Blood DNA methylation patterns are highly predictive of prenatal exposure to smoking, and differential methylation has been associated with maternal alcohol use. We extended this to determine whether DNA methylation patterns in cord blood are associated with prenatal exposure to opioids, cannabis, and polysubstance use. We also evaluated whether DNA methylation patterns have predictive utility. We examined 932 mother–child pairs in the Boston Birth Cohort between 1998 and 2020 with cord blood DNA methylation data. For each substance self-reported within 72 hr after birth, we performed an adjusted linear regression analysis at 865,859 CpG sites to identify related methylation differences. We generated polyepigenetic scores using summary statistics for each exposure and assessed predictive ability using cross-validation and receiver operating characteristic curves. Specificity of methylation associations was evaluated by assessing overlap across exposure summary statistics and using logistic regression for methylation scores, adjusted for concurrent use. We identified methylation changes at 72, 21, and 1 CpG suggestively associated with prenatal exposure to opioids, cannabis, and polysubstance use, respectively (*p* < 1e-6), in cord blood, reported for the first time for these exposures. We identified two loci associated at epigenome-wide significance with opioids and one with polysubstance use (*p* < 1e-8). Methylation scores were highly predictive and exposure-specific, with area under the curve accuracy of 91% for opioids, 90% for cannabis, and 93%–98% depending on polysubstance number. These CpGs provide biologic insights for reducing the impact of substance exposure, and these findings may serve as a biomarker of prenatal substance exposure for future studies and potential clinical utility.

## Introduction

Prenatal exposure to substances, including tobacco, alcohol, cannabis, opioids, and other illicit drugs, have known associations with poor health outcomes, including preterm birth [1–5], small for gestational age [6,7], and neonatal abstinence syndrome (NAS) [8–10], among others [11–16]. There is considerable interest in evaluating the effects of prenatal substance use exposures on health outcomes across the life course; however, they are often limited by a lack of robust exposure measures. Substance exposures are particularly challenging to collect in epidemiology studies. (1) Substance use during pregnancy is heavily stigmatized if not criminalized, and researcher-administered questionnaires may result in exposure misclassification [17,18]. (2) This information may not have been collected at all, limiting the ability of later researchers to use older cohort data. (3) While several toxicological biomarkers have been designed for the measurement of individual substances (such as cotinine for tobacco [19] and ethanol for alcohol [20]), they are limited by the short half-life of these metabolites in circulation. DNA methylation patterns have shown promise as a long-lasting biomarker for prenatal exposure across the life course. In addition to its potential biomarker utility, DNA methylation could provide a biologic mechanism for the effect of prenatal substance exposure on child health outcomes. CpG loci showing changes in neonates related to prenatal exposures could provide intervention targets and biologic insights to mitigate the impact of prenatal substance exposures on child health.

Over the past decade, literature has emerged showing that robust changes in DNA methylation are associated with prenatal exposure to smoking, including some that persist from birth through adulthood, independent of postnatal and personal smoking history [21–27]. More recently, genome-scale screens used to identify DNA methylation patterns in neonates were associated with prenatal exposure to other substances including alcohol via cord blood [23].

Cord blood is easily collected and reflects metabolic transfer to the fetus (rather than only maternal exposures). However, there remains a dearth of studies focused on other substances in cord blood, including opioids and cannabis, despite a rise in their use including during pregnancy. In 2009, 1.6 per 1,000 live births in the US were diagnosed with NAS or neonatal opioid withdrawal syndrome (NOWS), stemming from opioid exposure during gestation. By 2016, this rate rose to 8.8 per 1,000 live births [28]. To date, only a handful of studies have investigated DNA methylation changes related to prenatal opioid exposures, all of which were candidate-gene-based [29–33] or in placenta [34,35]. Notably, methylation effects have been demonstrated in the CYP2D6, OPRM1, and ABCB1 genes (among others) whether considering illicit opioid exposure, methadone (a full opioid agonist), or buprenorphine (a partial opioid agonist) exposure [32,36,37], likely due to the shared mechanism of opioid receptor pathways. While the placenta is an important reflection of the maternal–fetal interaction, it may not reflect what is actually transferred to the fetus—there is evidence that cell types and DNA methylation differ based on placental tissue facing the mother, the fetus, or in the middle between the two [38]. The placental and cord blood methylations are generally not strongly correlated and represent different organs, both of which are important to characterize as potential biomarkers [39].

Cannabis is also an increasingly common substance used by people during pregnancy, rising from 2.8 women with cannabis use disorder (CUD) per 1,000 deliveries in 2001 to 6.9 women with CUD per 1,000 deliveries in 2012 [40]. There is evidence that cannabis use is associated with differential methylation in genes hypothesized to affect brain function among adults [41,42]; however, whether there are similar patterns in children is unknown. Lastly, exposure to more than one substance, i.e., polysubstance exposure, has not been taken into consideration in prior studies, despite evidence showing that polysubstance use is common among pregnant people [43–45].

Here, we use a racially diverse, low-income birth cohort with unified substance exposure information and cord blood, genome-wide DNA methylation data to overcome prior limitations. We identify novel CpG changes associated with cannabis and opioid exposure prenatally and develop an accurate and substance-specific DNA methylation score for prenatal maternal use of opioids, cannabis, or polysubstance categories more broadly that, if further confirmed, can be applied to extant or newly-generated cord blood DNA methylation data to capture information about prenatal substance exposures.

## Methods

### Study participants

This study used data from the Boston Birth Cohort (BBC), which has been described in detail elsewhere [46]. In brief, the BBC is a longitudinal birth cohort that enrolled pregnant participants who delivered at Boston Medical Center (BMC), the largest safety net hospital in New England, from 1998 to the present. Participants and their children were recruited within 24–72 hr after delivery, and a baseline structured questionnaire was administered by trained research staff. The questionnaire collected information about sociodemographic characteristics, environment, and nutrition during pregnancy, social support and social assistance programs, and substance use history. Cord blood biospecimens were also collected at delivery. Both mother and child were followed prospectively via their electronic medical records (EMRs) at BMC until the child was 21 y old.

### Prenatal substance exposure

We derived a dichotomous prenatal substance exposure (yes/no) variable for each of three main substances of interest, including: opioids, cannabis, and polysubstances. Cannabis exposure was defined as ‘yes’ if the mother indicated use at any point in pregnancy on the baseline questionnaire. Prenatal opioid exposure was defined as ‘yes’ if either the mother self-reported use baseline or if the child was diagnosed with NOWS or NAS in their BMC electronic medical record, recorded via the International Classification of Diseases (ICD), with an ICD-9 code (779.5) or ICDs-10 code (P96.1). Indication of methadone use was also considered exposure for the purposes of this study; while patients prescribed methadone receive the necessary treatment, methadone is an opioid agonist that has been shown to have a similar biological mechanism of action. Prenatal tobacco exposure was defined as either maternal self-report or metabolic indication from urine cotinine-level measures taken at study enrollment. Given social desirability bias, which would bias self-report towards the null, a positive indicator in either metric resulted in being categorized as likely exposure. Cotinine levels ≥1.042 were considered ‘exposed,’ as determined by an adjusted receiver operating characteristic (AUROC) curve that maximized accuracy against self-report, as reported in prior BBC analyses [47]. Prenatal alcohol exposure was determined if the mother indicated use at any point during the pregnancy on the baseline questionnaire. Polysubstance exposure was defined across two categories: using two or more of the four substances (tobacco, alcohol, cannabis, and opioids) and using three or more of the four substances.

### Covariates

We included the following covariates from the maternal or child health records in our analyses: maternal education, race, ethnicity, pre-pregnancy body mass index (BMI), number of perinatal care visits, parity, child gestational age, and child sex.

Major maternal covariates of interest in our analyses included education, race, ethnicity, pre-pregnancy BMI, number of perinatal care visits (as a proxy measure of overall support during the pregnancy, which has been shown to be associated with maternal mental and physical health and more beneficial health behaviors during pregnancy [48,49]), and parity. All maternal variables were defined using self-reported data. Maternal education was defined as follows: (1) not completed high school, (2) completed high school, (3) begun but not completed college, and (4) completed college or higher. Maternal race and ethnicity were grouped as follows: non-Hispanic Black, non-Hispanic White, and Hispanic. No participants in our analytic sample reported other races or ethnicities. Maternal pre-pregnancy BMI was defined by taking the weight in kilograms and dividing by height in meters squared. Parity was defined as nulliparous (no live births) or multiparous, and the number of perinatal care visits was categorized into having 0, 1–2, 3–4, and 5 or more. Continuous maternal age at delivery was included as a covariate and obtained from EMR data obtained at delivery.

Child covariates were defined using EMR data obtained at delivery. We assigned a child as ‘preterm’ if the medical record indicated the delivery occurred at less than 37 gestational weeks of age; otherwise, they were defined as ‘term.’ Child sex was recorded at the time of delivery and assigned as ‘male’ or ‘female.’

### DNA methylation measures

Cord blood biospecimen collection and a detailed description of DNA methylation measurements for the BBC have been previously published [50]. Briefly, a total of 963 child cord blood samples were obtained by nursing staff following delivery. DNA samples were isolated using EDTA-treated peripheral white blood cells and underwent genome-scale methylation measurements using the Illumina Infinium MethylationEPIC BeadChip (850K) at the University of Minnesota Genomics Center (Supplementary Figure S1). We used the *minfi* Bioconductor package [51] and R version 4.1.3 to load, clean, and process DNA methylation array data (Supplementary Methods, Supplementary Figure S2). For each sample of the initial 865,859 CpG loci tested, files were assessed for bisulfite conversion, extension, hybridization, specificity, and additional factors. By comparing the median methylated and unmethylated signals for each array, outlier samples with median log2 intensity less than 10 were removed. There were 21 duplicated samples for quality control that were also removed from the sample. Sample-wise missing rates for the probes were calculated, with samples missing more than 2% of the probes removed. We applied normal-exponential out-of-band normalization (*noob*) and *ComBat* to remove batch effects [52,53]. Cell-type heterogeneity was then estimated using the *minfi estimateCellCount()* function [52] and the Flow Sorted Cord Blood reference panel [54]. This reference panel was derived from primarily European-ancestry American samples. Other reference panels were considered [54], but either were derived from other countries while still maintaining nearly all European ancestry or represented Asian-ancestry samples, which our sample notably did not have. In the light of this, we determined that this reference panel was most appropriate for our analysis despite limitations in ancestry diversity. Ancestry was estimated using DNA methylation within 10 base pairs of ancestry-informative SNPs, as described previously [55]. We further conducted surrogate variable analysis (SVA) to control for additional factors, such as batch effects and other confounding factors, such as maternal age and ancestry-related confounding. In the SVA model, we also included the other substances in each of our EWAS, thereby implicitly adjusting for the influence of concurrent substance use in our substance-specific EWAS. We did not include other substances in our polysubstance models, as this would amount to over-adjustment of the main effect of interest – specifically, the compounding effect of multiple substances. This was performed using the *sva* Bioconductor package using the Leek method, which then generated an elbow plot to identify where the drop in eigenvalues occurred out of all values suggested by the Leek method for its suggested number of variables [52]. Given the Leek method can generate a greater number of surrogate variables compared to more conservative approaches, this allowed further refinement to ensure adequate control of confounding without overestimation.

While SVA has been shown to be robust in adjusting for these factors, we further adjusted for the ancestry-based principal components, cell-type heterogeneity, and maternal self-reported education, as this was the only self-reported confounder to continue to be associated with our exposure and outcome after adjusting for these prior other factors, to minimize confounding. We retained European-ancestry participants despite a small sample size in an effort to maximize power and provide some comparability to prior research which has centered historically on European-ancestry samples exclusively. Additionally, using these methods to account for ancestry, there was not evidence of ancestry-related confounding in our diagnostic tests, which provides further support that the inclusion of this small portion of primarily European ancestry participants did not meaningfully impact results due to population stratification alone.

### Epigenome-wide discovery analysis (EWAS)

We used the *lmFit()* function in the *limma* Bioconductor package [56] to identify CpG loci, where DNA methylation levels were associated with each of three prenatal substance exposures, including opioid, cannabis, and polysubstance use. We did not perform an EWAS for either prenatal smoking or alcohol exposures because larger consortia-level studies have already been conducted [21,23]. The lambda inflation factor was calculated and the observed versus expected *p*-values were plotted on a quantile–quantile plot. In the final epigenome-wide association models using beta values of DNA methylation, 12 surrogate variables, maternal education, 10 principal ancestry components, and all cell-type estimates were included in the statistical model applied to each prenatal exposure. We used a liberal adjusted *p*-value threshold for exploratory analyses of 1e-6 and a more stringent adjusted *p*-value threshold of 1e-8 correcting for multiple testing to determine a statistical significance [57]. Stratification by likely ancestry group was not possible due to a lack of power—for example, when stratifying likely African ancestry children from the sample, only two participants had reported opioid exposure during gestation, which is underpowered to determine an effect. Therefore, we included SVs and PCs to both adjust for potential population stratification and conducted analyses within the full sample.

### Dnam score derivation and analysis

To assess the ability of child cord blood DNA methylation patterns to predict prenatal exposure to each substance, we built methylation exposure scores (an aggregate measure of exposure associated CpGs across the genome). We applied a train/test approach at multiple EWAS *p*-value thresholds to identify an optimal *p*-value cutoff for deriving the final methylation scores to use for prediction. As shown in Supplementary Figure S3, we split our BBC dataset into 90% training and 10% testing sets. In the 90% training dataset, we conducted k-fold cross-validation (k = 4) using 75% of the training data in each iteration to generate the EWAS summary statistics and DNAm scores for each *p*-value threshold cutoff examined (1e-7, 1e-6, 1e-5, 1e-4, 1e-3, 1e-2, 1e-1, 1). We then tested the performance of each DNA methylation score on 25% of the data left out in each respective iterations (e.g., 25% of the 90% training dataset). Once a final optimal *p*-value cutoff was identified, we applied the summary statistics from a total of 90% training sets to 10% independent testing sets to generate unbiased estimates of the area under the curve (AUC) estimates and Nagelkerke’s R^2^ [58].

DNA methylation exposure scores were generated using the epigenome-wide association study (EWAS) summary statistics from the BBC training dataset for cannabis, opioid, and polysubstance (defined in two ways: ≥2 substances and ≥3 substances). For a given individual as follows:

$$\mathrm{Methylation}\;\mathrm{Score}=\overset{n}{\underset{i=1}{\sum }}\frac{\beta_{1,i}}{\overset{ˉ}{\beta }}*\left[\mathrm{Cp}G_{i}-\mathrm{mean}\left(\mathrm{Cp}G_{\mathrm{unexposed}}\right)\right]$$

where $\frac{\beta_{1,i}}{\overset{ˉ}{\beta }}$ is equivalent to the effect size of a CpG divided by the average effect size of all CpGs. We subtracted the average methylation of all CpG loci among unexposed samples from the CpG methylation percentage for each locus. We applied this standardizing and centering approach to reduce the influence of outlier CpGs with extreme effect estimates and produce a score that remains interpretable as a relative departure from unexposed-like methylation patterns, even when comparing across multiple EWAS through our cross-validation measures.

### Specificity analyses and enrichment

We evaluated the specificity of the methylation changes we identified for each substance in two ways. First, we counted the number of loci identified in our EWAS for opioids, cannabis, and polysubstance exposure individually that overlapped with loci identified for each of the other substance exposure groups and also with CpG loci identified through prior epigenome-wide DNA methylation studies of tobacco [21,22] and alcohol [23]. Second, for DNA methylation exposure scores, we tested the association between the exposure methylation score and self- or medical record-based prenatal exposure while adjusting for use of other substances (e.g., when estimating associations with cannabis use, we also controlled for using opioids, smoking tobacco, or consuming alcohol in the model) via generalized regression models. Additionally, we conducted EWAS for tobacco smoking (defined as any smoking during pregnancy or exposure confirmed by cotinine at the time of delivery) and alcohol consumption (defined as any alcohol consumption reported during pregnancy) using the same methods as for our opioid, cannabis, and polysubstance EWAS to determine whether there was overlap with previously reported EWAS in cord blood. Finally, we conducted eFORGE 2.0 analyses for the top 100 CpG loci for all four substance exposures to elucidate tissue- and cell-type specific signals and their functional relevance within 1kbps of loci using a false discovery rate (FDR) q-value threshold of 0.05 for significance over 1,000 repetitions.

### Human subject protection statement

Written informed consent was obtained from all the participating mothers before their enrollment at the BBC. The informed consent, protocol, and data collection instruments were reviewed and approved by the institutional review boards (IRB) of the BMC and Johns Hopkins Bloomberg School of Public Health under IRB00022869. This study adhered to ICMJE guidelines on the Protection of Research Participants, The Belmont Report, and the Declaration of Helsinki and their relevant updates. The privacy rights of human participants have been observed, including obtaining an NIH certificate of confidentiality. Per best practices to maintain participant confidentiality, we report any subgroups with less than five participants as simply ‘*n* < 5’ in tables.

## Results

After applying sample- and probe-level filters, a total of 865,859 CpG sites and 932 samples were included in our analytic sample (Supplementary Figure S1 and S2). Of these, 251 (26.9%) had prenatal exposure to any one substance (opioid, cannabis, polysubstance, alcohol, or tobacco; Table 1). The most reported was tobacco smoking, which represented 21.9% of our overall sample (*n* = 204). Alcohol was reported among 8.3% (*n* = 77), and cannabis was reported among 3.5% (*n* = 33) of mothers. Opioid exposure was present in 6.4% of the samples (*n* = 60). Prenatal polysubstance exposure for two or more of these substances was present in 6.5% (*n* = 61) of children, and exposure to three or more substances was present in 0.8% (*n* = 8) of our overall sample. Most maternal participants reported non-Hispanic Black race/ethnicity (70.8%, *n* = 660), followed by Hispanic (23.7%, *n* = 221) and non-Hispanic white (5.5%, *n* = 51). At birth, 493 (52.9%) of our sample were identified as male, and 439 (47.1%) as female. Information on prevalence trends by year is presented in supplements (Supplementary Figure S2), demonstrating no major temporal trends between years based on exposure. Additional information for use during specific trimesters is presented in supplements, but cell sizes were small for certain substance exposure groups, and there was no sufficient power to stratify by trimester of use. There was an overall trend of greater prevalence of use during trimester 1 compared to later trimesters (Supplementary Figure S3) that was statistically significant via Fisher’s exact test for cannabis and opioid exposure (*p* < 0.05).

**Table 1.** Descriptive statistics of maternal and child characteristics in our analytic sample.

	Exposed to opioids *N* = 15	Exposed to cannabis *N* = 33	Exposed to polysubstances (2+) *N* = 65	Exposed to any substances *N* = 251	Not exposed to substances *N* = 681
Maternal age—Median [IQR]	31.24 [27.07, 32.36]	24.38 [21.26, 31.37]	28.15 [24.38, 33.21]	26.43 [22.22, 31.55]	28.67 [23.26, 33.37]
Maternal education					
Completed primary school	0	0	0	*n* < 5	23 (3.4%)
Some high school	*n* < 5	14 (42.4%)	19 (29.2%)	79 (31.5%)	158 (23.2%)
Completed high school	8 (53.3%)	12 (36.4%)	28 (43.1%)	110 (43.8%)	251 (26.9%)
Some college	*n* < 5	6 (18.2%)	14 (21.5%)	48 (19.1%)	155 (22.8%)
Completed college	*n* < 5	*n* < 5	*n* < 5	12 (4.8%)	94 (13.8%)
Maternal race/ethnicity					
White	10 (66.7%)	*n* < 5	11 (16.9%)	31 (12.4%)	20 (2.9%)
Black	*n* < 5	26 (78.8%)	42 (64.6%)	180 (71.7%)	480 (70.5%)
Hispanic	*n* < 5	5 (15.2%)	12 (18.5%)	40 (15.9%)	181 (26.6%)
Child’s Sex					
Male	9 (60.0%)	13 (39.4%)	34 (52.3%)	122 (57.3%)	371 (51.6%)
Female	6 (40.0%)	20 (60.6%)	31 (47.7%)	91 (42.7%)	348 (48.4%)
Preterm birth					
Yes	7 (46.7%)	12 (36.4%)	21 (32.3%)	58 (23.1%)	112 (16.5%)
No	8 (53.3%)	21 (63.6%)	44 (67.7%)	193 (76.9%)	568 (83.5%)
Parity					
Nulliparous	7 (46.7%)	19 (57.6%)	41 (63.1%)	112 (44.6%)	307 (45.1%)
Multiparous	8 (53.3%)	14 (42.4%)	24 (36.9%)	139 (55.4%)	374 (54.9%)
Yearly income					
≤$35,000	7 (46.7%)	16 (48.5%)	35 (53.8%)	129 (51.4%)	314 (46.1%)
>$35,000	8 (53.3%)	14 (42.4%)	27 (41.5%)	96 (38.2%)	283 (41.6%)
Don’t Know	0	*n* < 5	*n* < 5	26 (10.4%)	84 (12.3%)
Maternal pre-pregnancy BMI—Median [IQR]	23.17 [22.09, 28.78]	23.87 [21.90, 27.91]	25.25 [22.27, 29.17]	26.29 [23.04, 31.07]	25.09 [22.26, 29.95]
Tobacco exposure	13 (86.7%)	14 (42.4%)	38 (58.5%)	93 (43.7%)	0
Alcohol exposure	3 (20.0%)	8 (24.2%)	38 (58.5%)	77 (36.2%)	0

Note: Cells with less than fve participants are written as ‘*n* < 5’ to provide greater protection to participant confidentiality. Given the small number of participants reporting using 3+ substances (*n* = 8), we are unable to report the distribution of these covariates in a meaningful way, as nearly all cells would be *n* < 5, and therefore are omitted from this table.

### DNA methylation associated with prenatal opioid exposure

**Figure 1.**
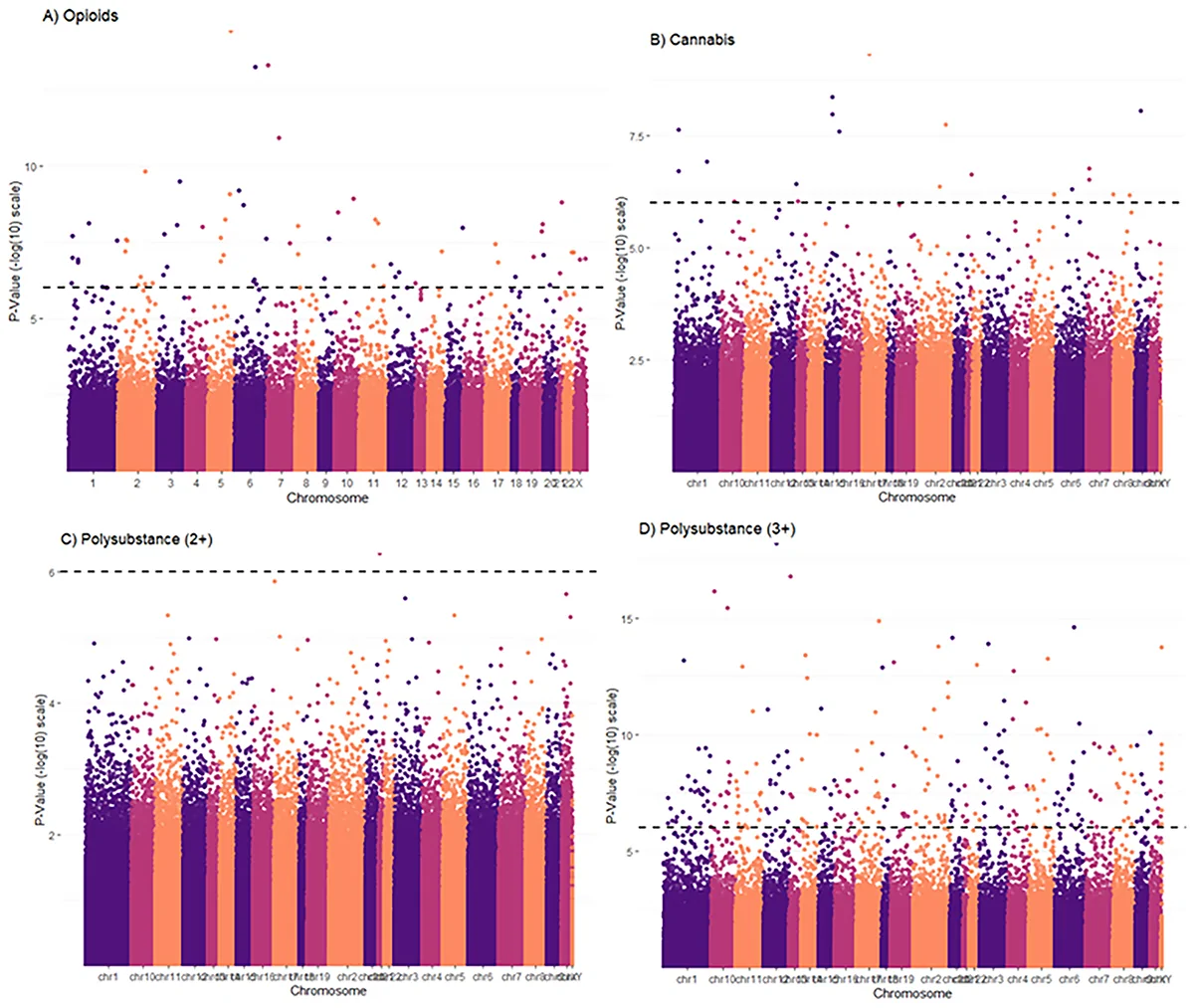
Manhattan plots showing the results of each epigenome-wide association screen for prenatal exposure to (A) opioids, (B) cannabis, (C) 2 or more substances, and (D) 3 or more substances.

Our genome-scale discovery screen identified two loci with epigenome-wide significance with our stringent threshold (adjusted *p*-value < 1e-8) and 72 loci with our liberal threshold (adjusted *p*-value < 1e-6) associated with prenatal opioid exposure (Figure 1). The lambda value is a statistic indicating inflation in the number of loci we tested that have low *p*-values beyond what we expect if our test statistics are properly distributed, and for the opioid EWAS our lambda value was 1.04 (Supplementary Figure S4), suggesting minimal inflation. The most significantly associated CpG site was cg11236288 in the *KCNIP1* gene, which was associated with a 3.78% reduction in DNA methylation among opioid-exposed neonates (Supplementary Figure S4).

Each point represents 1 CpG site examined. The x-axis shows the genome location of each CpG. The y-axis shows the negative log 10 *p*-value. The horizontal dashed line denotes the suggestive genome-scale *p*-value significance threshold after adjusting for multiple testing. Coloring is to distinguish between different chromosomes and does not represent additional interpretation.

### DNA methylation associated with prenatal cannabis exposure

For prenatal cannabis exposure, methylation changes in 21 child cord blood CpGs achieved liberal significance (*p* < 1e-6), and none were associated at the stringent threshold (Supplemental Table S5). The top CpG site was cg0670361, which was associated with a 2.5% reduction in DNA methylation in the *RP11.78O7.4* gene. The next most significantly associated CpG sites were cg10426464 in the *C2CD4B* gene and cg13949487 in the promoter region of the *ERCC6L2* gene.

### DNA methylation associated with polysubstance use

We assessed differences in DNA methylation in child cord blood related to maternal use of two or more substances during pregnancy, i.e., polysubstance use, compared to single or no substance exposure. One significant locus at cg07392829 on chromosome 21 in the *KRTAP6-3* gene (Supplemental Table S6) was identified with the liberal (1e-6) but not stringent threshold (1e-8). The lambda value for polysubstance use of two or more substances was 0.99 (Supplemental Table S4). We also searched for methylation associated with prenatal exposure to three or more substances and observed inflation was slightly higher, with a lambda value of 1.06. In total, 303 loci were statistically significant at the genome-wide level for this analysis (Supplemental Table S7). The most significantly associated locus was the cg12651615 site in the *HELB* gene, which had a *p*-value of 6.05e-19 and was associated with a 4.02% reduction in DNA methylation. The cg07392829 site was not significant when defining polysubstance use as more than three substances (Supplemental Table S7); however, the magnitude of the DNA methylation difference and direction of effect were similar (1.81% reduction for two or more substances, vs. 0.96% reduction for three or more substances). Finally, we also report associations with tobacco and alcohol in Validity EWAS for tobacco and alcohol.

To interrogate the robustness of these findings compared to other benchmarking studies, we also conducted EWAS for tobacco and alcohol exposure during pregnancy, which we present in Supplemental Tables S8 and S9.

### Overlap of loci associated with each prenatal exposure

**Figure 2.**
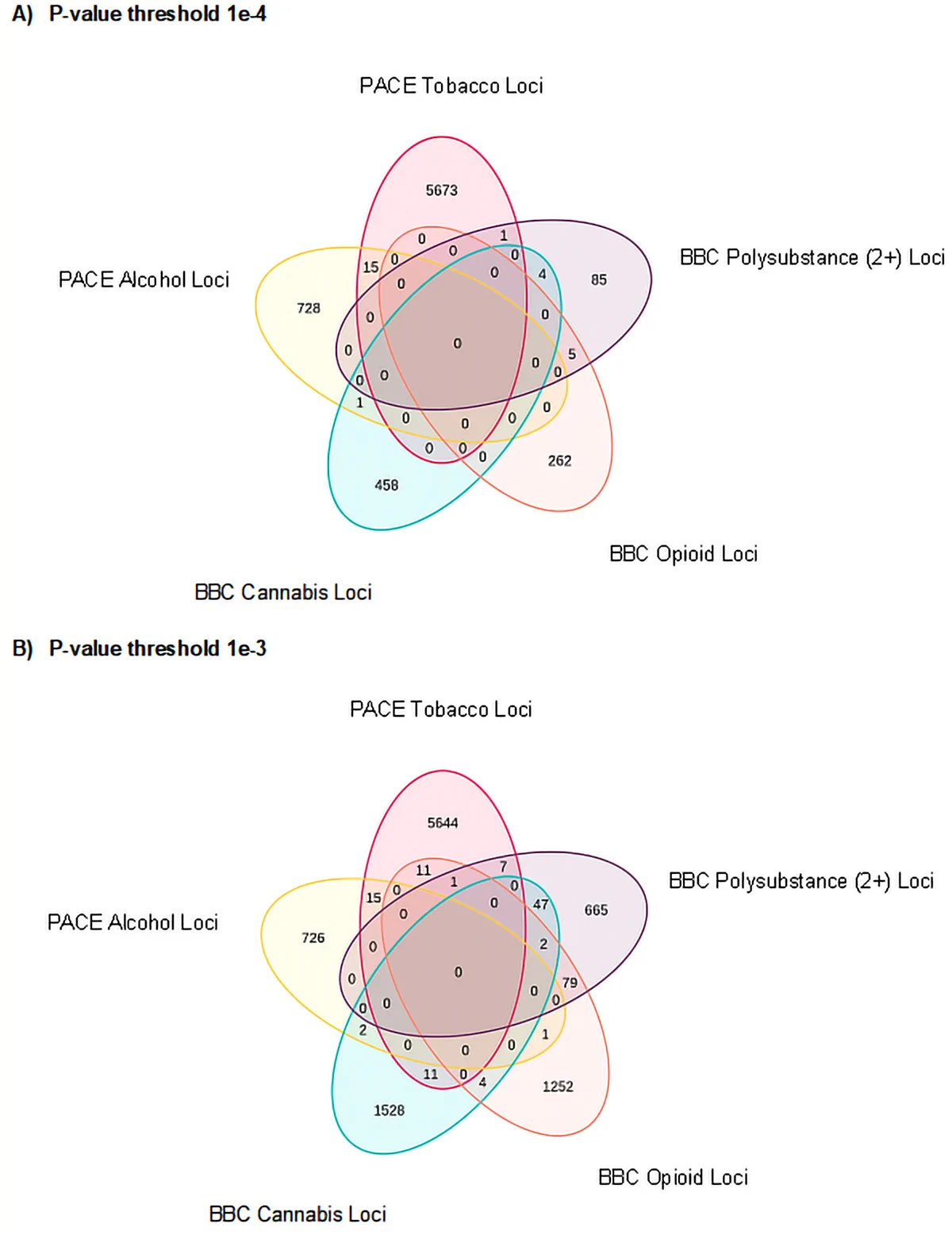
Venn diagram of overlapping CpG sites by substance exposure for (A) *p*-value threshold for opioids, cannabis, and polysubstance use (2+) of 1e-03 in the BBC and (B) *p*-value threshold of 1e-04 in the BBC.

Note: PACE loci refer to loci derived from the Pregnancy and Childhood Epigenetics (PACE) consortium and included all epigenome-wide significant loci previously identified. BBC loci refer to loci derived from epigenome-wide association analyses in the BBC and only includes CpG sites with *p*-value less than the specified cutoff. Panel A: Venn diagram of overlapping CpG sites using a *p*-value threshold <0.0001 in the BBC. Panel B: Venn diagram of overlapping CpG sites using a *p*-value threshold of <0.001 in the BBC. Polysubstance use was defined as self-reported using two or more of any substances.

**Figure 3.**
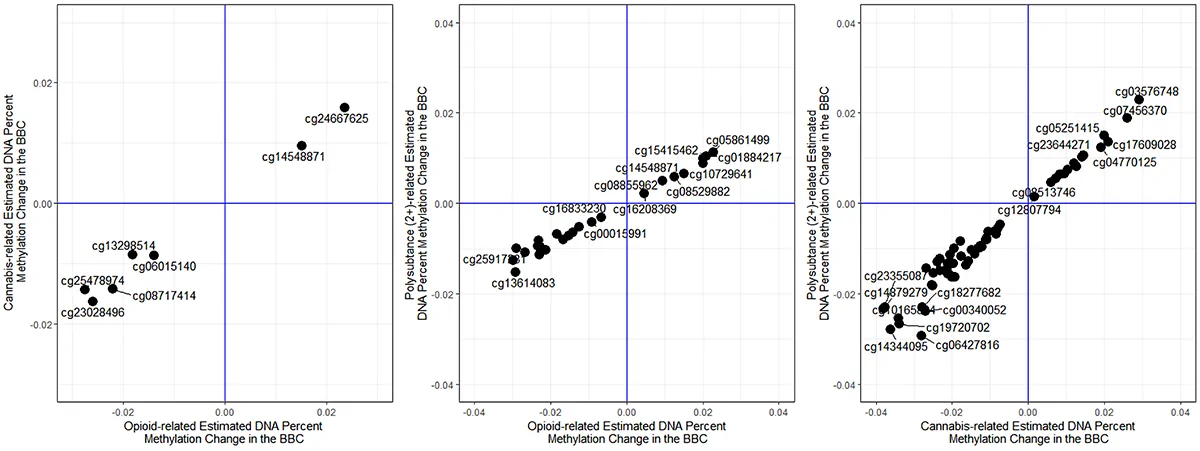
Quadrant plots of estimated DNA methylation changes associated with opioids, cannabis, and polysubstance (2+) exposure in the BBC for overlapping loci, demonstrating concordant or discordant direction of effect for multiple substance exposures.

Using a *p*-value threshold of 1e-3, the results from our opioid and cannabis screens had seven loci in common, four of which were specific to cannabis and opioids only, one of which was also present in the PACE alcohol analysis, and two of which were also associated with the polysubstance (2+) exposure (Figure 2). All of the seven loci were in the same direction of effect between their respective substances (Figure 3). We also compared our opioid and cannabis results from the BBC with external consortia results for CpGs showing methylation changes associated with prenatal smoking exposure. A total of 11 CpGs overlapped between the smoking results and prenatal exposure to opioids, and a separate 11 were shared with the cannabis EWAS, using a 0.001 *p*-value threshold, with no overlap. Exposure to two or more substances shared methylation changes with opioid exposure at 79 CpGs (10.1% of the 783 loci associated with exposure to 2+ substances at the threshold) and with cannabis at 47 CpGs (6.0% of the 783 loci) though this still reflected a minority of the overall number of associated CpG sites for the polysubstance EWAS.

Quadrant plots report a log2-fold change in DNA methylation between exposed vs. unexposed participants, associated with opioid, cannabis, and polysubstance (2+) exposure among CpGs with suggestive associations in both substances.

### Methylation scores for prenatal substance exposure classification

**Figure 4.**
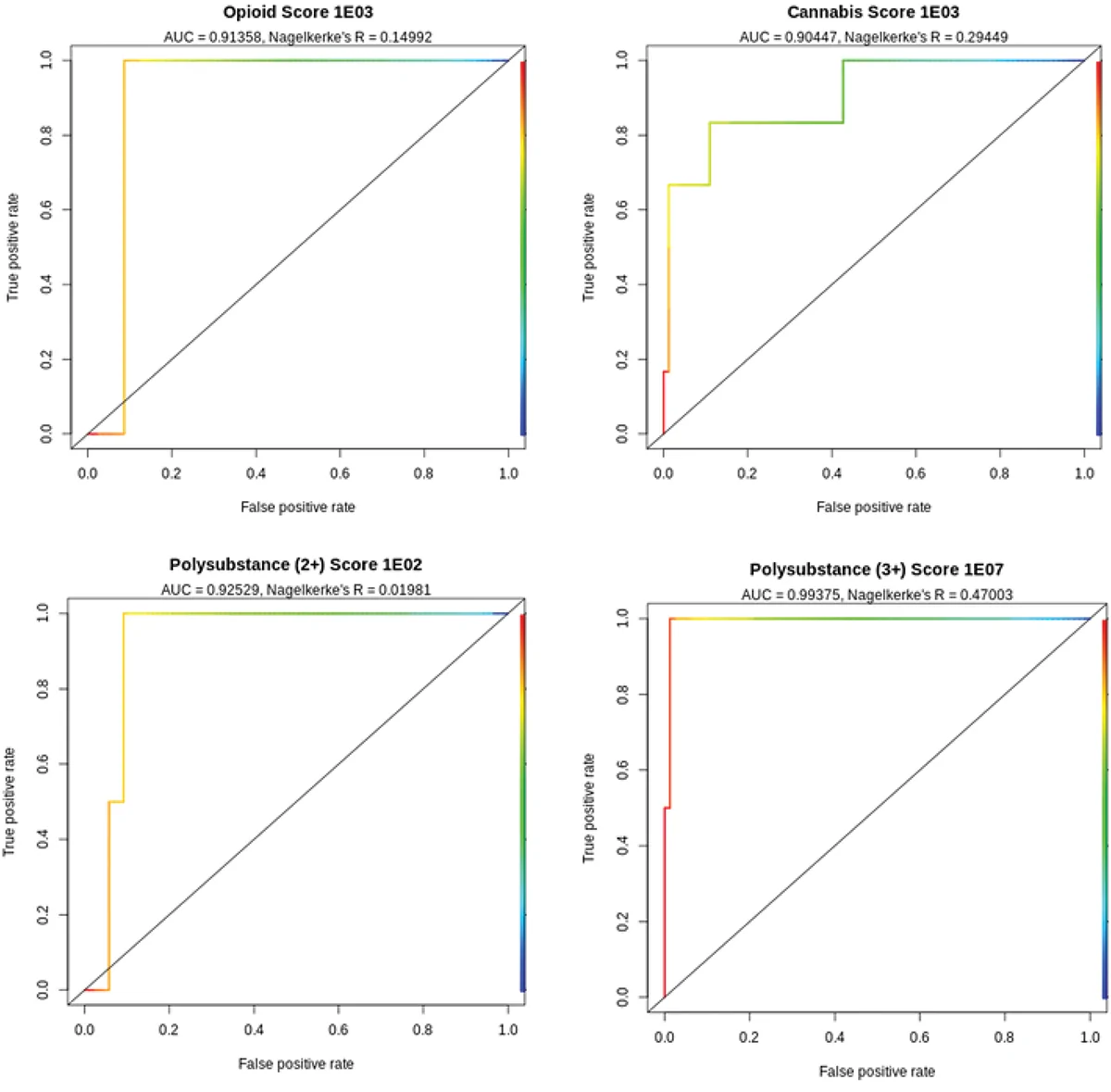
Receiver operator curves (ROC) reporting the ability of DNA methylation exposure scores from child cord blood samples to accurately capture prenatal exposure to opioids, cannabis, and polysubstance exposure.

Generating the methylation score and testing in the 90%–10% split dataset, we had high AUC across all samples. The highest was opioids, with an AUC of 0.91 (Nagelkerke’s R2 = 0.15) (Figure 4). Cannabis also displayed a high discrimination rate with an AUC of 0.90 (R2 = 0.28). Finally, for polysubstance exposure, defined as using two or more substances, there was an AUC of 0.93 (R2 = 0.02), and for three substances or more, there was an AUC of 0.99 (R2 = 0.47).

False positive rate is shown on the x-axis and false negative rate on both y-axes, shown on either side. AUC and Nagelkerke’s R^2^ are also reported. *p*-value cut-off thresholds were determined to maximize AUC, and the best-performing cut-off is reported in the panel titles.

**Figure 5.**
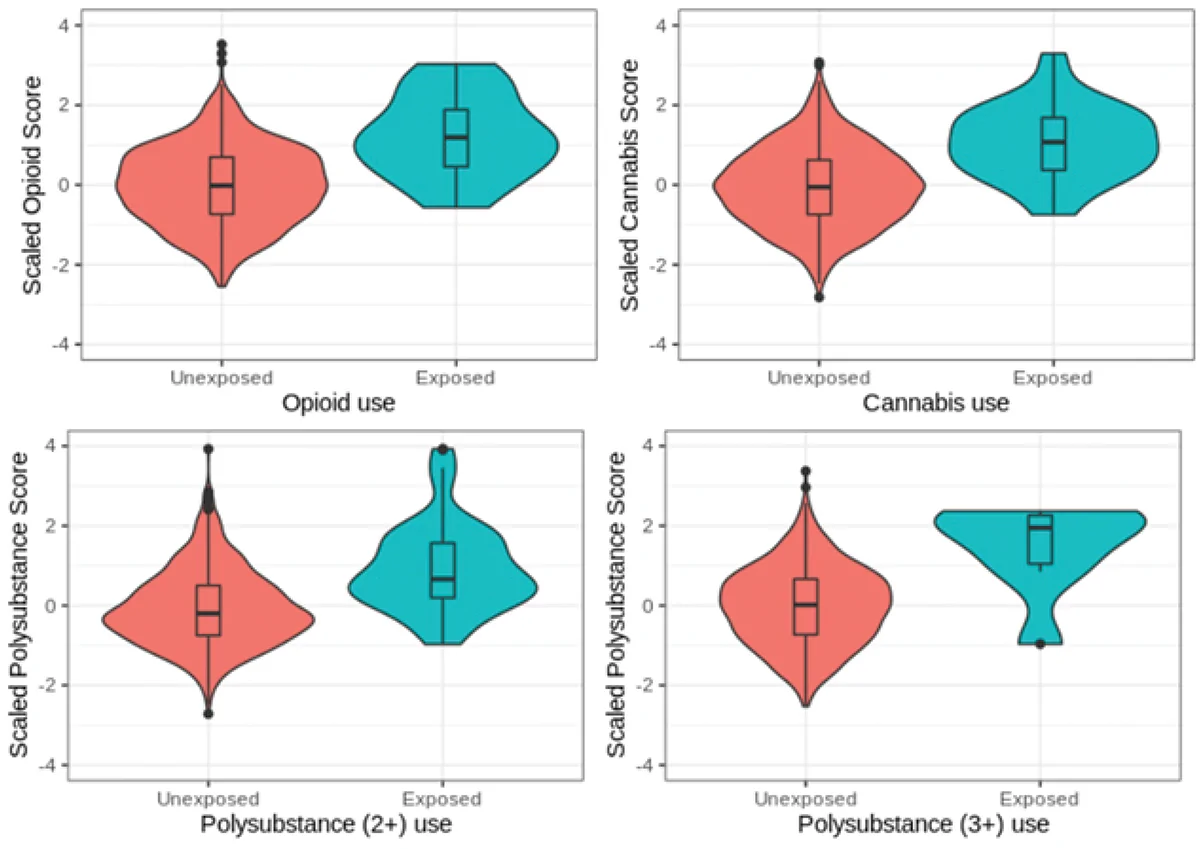
Distribution of scaled polyepigenetic scores among exposed and unexposed samples for opioids, cannabis, and two polysubstance categories.

We then tested for association between each substance methylation score with the respective substances, while controlling for concurrent use of other substances to assess whether the methylation scores captured differences related to other substances or were specific. Overall, the scores were highly significantly associated with the respective substances (Figure 5) and remained associated following successive adjustments for other substances (Table 2). It is worth noting that given the small number of participants with 3+ substance exposure, truncation occurs for the score distribution among those exposed, and people with especially high scores beyond 2.5 standard deviations (representing the top 0.62% of this group) above the mean are not represented in our data. The opioid scaled methylation score was affected the most by controlling for alcohol exposure. After an initial association of 5.78 odds of opioid exposure (95% CI: 1.68, 27.72), the estimated OR became 3.39 (95% CI: 2.02, 5.90) after adjusting for alcohol exposure; however, the confidence intervals overlapped with the unadjusted estimate. When adjusting for all other substance exposures, the epigenetic score was associated with 4.69 odds of opioid exposure (95% CI: 2.44, 9.81) and the cannabis epigenetic score was associated with 3.48 (95% CI: 2.26, 5.57) odds of exposure. When examining the association between the opioid and cannabis polyepigenetic scores and other substance exposures, there were no significant associations (Supplemental Table S10). The Spearman rank correlation matrix is also provided (Supplemental Table S11). Finally, eFORGE charts (Supplemental Figures S9 – S12) identified primarily embryonic stem cells, induced pluripotent stem cells, and fetal tissue types associated with these differentially methylated loci, though none of these were significant at the 0.05 level.

Scores reported were generated using the best-performing cut-offs. Scaling was done by centering at the mean and dividing by the standard deviation.

**Table 2.** Associations with respective substance use and methylation exposure scores, while controlling for exposure to other substances.

	Association without controlling for other substances OR (95% CI)	Association controlling for opioids OR (95% CI)	Association controlling for cannabis OR (95% CI)	Association controlling for tobacco OR (95% CI)	Association controlling for alcohol OR (95% CI)	Association controlling for all OR (95% CI)
Scaled opioid score	5.78 (1.68, 27.72)	NA	3.45 (2.05, 6.02)	4.64 (2.42, 9.63)	3.39 (2.02, 5.90)	4.69 (2.44, 9.81)
Scaled cannabis score	3.14 (2.17, 4.66)	3.15 (2.17, 4.67)	NA	3.44 (2.25, 5.48)	3.16 (2.16, 4.73)	3.48 (2.26, 5.57)

OR: Odds ratio; 95% CI: 95% Confidence Interval.

## Discussion

We identified novel cord blood methylation differences at CpG sites in neonates associated with prenatal opioid and cannabis exposures, evaluated overlap of CpGs across substances, and built highly accurate and substance-specific classifiers to predict prenatal exposure to each substance from cord methylation data. The CpGs we identified provide biologic targets for future work to evaluate the relationship between DNA methylation and child health outcomes as a possible mechanism for prenatal substance effects on child health. Most CpGs we identified appear to be substance-specific and those that did overlap had similar directions of effect. The methylation scores may be useful as a potential biomarker for substance exposure during gestation. This may be very useful for epidemiologic studies of prenatal substance exposure, where obtaining actual substance exposure information is challenging. We emphasize that our findings do not predict future substance exposure or neonatal effects using DNAm measures but rather identify a potential biomarker or associated endophenotypic sequelae after such exposure has occurred during gestation. This is the largest investigation to date of neonatal cord blood methylation associated with opioid exposure [59], and one of the first to investigate cannabis and polysubstances, and may be useful in identifying patients who may be at greatest risk for substance exposure-related biological sequelae and who may benefit from additional resources or clinical support.

While we ultimately had a small sample, this study, to our knowledge, is the largest number of participants with prenatal opioid exposure to date. Other studies of prenatal opioid exposure have been in placental tissue and included 32 and 19 exposed participants. This investigation identified both novel loci not previously reported as associated and validated known loci. In our EWAS study for opioid exposure, we found the most significant association with methylation changes at a CpG located in the *KCNIP1* gene, which encodes the potassium voltage-gated channel interacting protein. Interestingly, DNA methylation changes in this gene have also been associated with depression among monozygotic twins [60] and multiple sclerosis based on post-mortem brain samples [61]. *KCNIP1* has also been identified in other neuropsychiatric disorders, such as attention-deficit hyperactive disorder (ADHD) [62], which is highly comorbid with substance use disorders [63]. This biological pathway related to *KCNIP1* and its expression likely has far-reaching implications across cellular function, but its importance for neurons lends credence to its potential as a biological pathway affected by prenatal substance exposures. Finally, in this EWAS, a subset of the loci have been examined in the Environmental influences on Child Health Outcomes (ECHO) cohort, which found significant associations between the cg14303187 loci in the *SHC3* gene and opioid exposure in a composite sample of 25 exposed and 360 unexposed participants [64]. This sample had fewer exposed participants than our report here, which may have less power, and this was an aggregate of multiple cohorts that may differ from our single cohort in the BBC, leading to different environmental influences that serve as unmeasured confounders between the samples. Additionally, a report of methadone-maintained mothers also identified a potassium voltage-gated channel gene (*KCNC1*) [59], which taken with our study, may suggest that this mechanism is particularly important for how opioid exposure influences children in gestation. Notably, we did not find major associations with DNAm loci in the OPRM1 gene, which has previously been investigated in candidate gene studies. This is in line with a recent 2025 placental tissue study of opioid exposure in gestation, that similarly did not replicate DNAm in this gene. This may reflect the importance of developmental processes on DNAm plasticity and reflect a difference in the biological pathway associated with gestational exposure versus exposure as an adult after many developmental processes have been resolved.

It is notable that it was not possible to replicate accuracy measures with the scores in this sample, as, in the ECHO consortium, which spans 69 cohorts, only 25 individuals were reliably identified as opioid-exposed with comparable cord blood DNAm data available that passed quality control measures, and even fewer who met criteria for polysubstance use (2+ and 3+). Accuracy measures would be highly volatile with even one birth being correctly or incorrectly classified and would create a biased impression of the external validity of these scores beyond this initial pilot study. Further work using specialized recruitment methods should be considered for cohorts interested in using similar scores and analyses.

Beyond this, additional loci in CACNA2D3 (cg08688543) was significant, which is associated with nicotine dependence [65] and habituation learning involved in calcium signaling [66]. Loci in PDCD4 (cg27408345), which regulates apoptosis and is commonly discussed in tumor suppression literature [67], AGPAT3 (cg17636538) is important in lipid metabolism and has been associated with intellectual disability [68], indicating some effects in the brain. NFE2L2 additionally plays a role in the antioxidant response and has been associated with mouse models of Alzheimer’s disease [69]. And finally, TTC7A was also associated with exposure, which is known to be a driver of gastrointestinal disease, immunodeficiency, and inflammation [70]. Taken together, these genes demonstrate potential far-reaching influences of exposure during gestation that may affect critical functioning and development in children across cellular functions both in the brain and beyond.

For prenatal cannabis exposure, we found notable CpG methylation associations, including two differentially methylated loci in the *C2CD4B* gene. *C2CD4B* has been identified as important to diabetes risk [71–74]. One recent study also implicated this gene in intracerebral hemorrhaging [75]. Therefore, DNA methylation changes in *C2CD4B* may be related to metabolic and vascular processes potentially impacted by cannabis exposure, though it is not clear what link there may be between cannabis and glucose metabolism [76,77]. The most comparable study to ours had 10 pregnancies with cannabis exposure, and 20 with cannabis-tobacco co-exposure during gestation and cord blood available among children in the Avon Longitudinal Study of Parents and Children (ALSPAC), with all European-ancestry participants. They also demonstrated epigenome-wide significant loci from birth, and longitudinal whole blood samples associated with differential methylation at later timepoints, including ages 15–17 in the ALSPAC cohort [78]. An additional study of US adults, which also focused on a multi-ancestry population, found significant associations at *ADGRF1, ADAM12, ACTN1*, and *LINC01132* [79]. While *LINC01132* was not associated in our sample, another gene of the same family (*LINC00476*) is associated with one locus of suggestive significance (cg13949487), which has been implicated in the suppression of non-small cell lung cancer tumor progression [80]. Major differences between our study and these include timing and ancestry components. Additionally, substance supply and use patterns notably differ between countries, and were recruited between 1991 and 1992, while our participants in the BBC were recruited between 1998 and 2021, representing potential differences in their exposure to cannabis products. To this end, while it was not possible to identify a clear delineation point based on temporal year and there was not a significant association on year alone when accounting for maternal age, we present the yearly breakdown of participants for reference, in supplement. Further studies to investigate the relationship between cannabis exposure, *C2CD4B* methylation, and cardiometabolic conditions are needed.

This is the first epigenome-wide association screening for prenatal polysubstance exposure to our knowledge. We found that the loci identified as associated with polysubstance use had minimal overlap with loci identified in single substance analyses. The most significant CpGs showing methylation changes related to exposure to three or more substances were located in the *HELB* (DNA Helicase B), which is involved in DNA damage response and unwinding of the DNA for replication and repair [78]. This suggests that polysubstance exposure impacts methylation in core biologic functional processes, which could lead to a number of downstream effects.

Although most of the loci we identified showed little overlap across substances, some showed methylation changes in the same direction for more than one substance. It is also possible that we were underpowered to detect some overlapping loci due to differences in the number of exposed individuals per substance. Overlapping loci imply that some of the methylation targets for prenatal substances may be shared and converge on the same biologic pathways. For example, cg25478974 located in the gene *NLRX1* was discovered to show a decrease in DNA methylation associated with prenatal exposure to opioids and cannabis independently of the liberal threshold. *NLRX1* is implicated in mitochondrial antivirus response, and in mouse models, has been shown to impact a wide range of diseases and disorders, including preterm birth, where it is expressed in the amnion, placenta, and choriodecidua [79,80]. Particularly in the light of its associations with preterm birth, which is also associated with substance exposure [1,2,4,11,12], it is possible that the immune response impacted by *NLRX1* expression mediates substance exposures and birth outcomes. Further research into this potential mechanism may yield greater insights. Additionally, in the eFORGE analyses, there were no statistically significant trends after adjusting for false discovery, however stem cell types showed the most association overall, which is somewhat non-specific. Additional associations were found in the fetal thymus for opioid EWAS hits, which may be linked to the fetal immune system, the fetal heart for cannabis and polysubstance 2+ EWAS hits, and the fetal intestine for polysubstance 3+ EWAS hits. Further analyses for cell-type expression using transcriptomic or RNAseq analyses may yield more specific findings in the future work that may better identify pathways.

Notably, stigmatization based on these early life biomarkers may be of concern [81]. Identifying pregnant people or children with these exposures based on cord blood may benefit epidemiologists and researchers, but for their widespread use in clinical settings, there must be safeguards to ensure that, if there is likely exposure to a substance, these families are met with resources rather than stigmatization or worse, law enforcement involvement [82]. Clinicians and researchers should consider this one marker among many that indicates a patient’s health risk, and the implications of false positives or negatives must be carefully considered in the future research.

Given known social desirability bias in self-reported substance use [83,84], especially during pregnancy [85,86], as well as the criminalization of substance use in pregnancy in some states of the US, there remain ongoing challenges when measuring substance use in pregnancy. This is present even when using self-reported measures designed specifically for the pregnancy period [87]. Direct measures of some substances are possible, but these are not commonly run and can miss earlier pregnancy exposures due to the short half-life of many substances. The presence and use of an empiric methylation biomarker present in the child can provide an important complementary tool to bolster more traditional exposure metrics or to overcome the lack of collection of substance exposures for use in epidemiology studies.

There are additional limitations to our study. While we leveraged our cohort study to maintain independence between our training and test datasets, overfitting of our models may still occur. Future analyses with larger sample sizes may also benefit from region-based aggregation approaches and examination of co-methylated regions, which could provide additional biological context beyond the individual CpG associations reported here. There are considerable challenges to assembling a study sample with sufficiently detailed substance exposure data and enough exposed participants to be powered for these analyses among birth cohorts with DNA methylation available. Therefore, replication in a fully separate population remains an important future consideration for generalizability.

To the best of our knowledge, this is the first study to examine and evaluate exposure to multiple prenatal substances in the same sample, including polysubstance-specific effects [88]. Most epigenome-scale studies to date for single substance exposures have largely focused on European ancestry populations [21,23,24,89,90], leaving significant data gaps for underrepresented minority and low-income populations in the US and around world. We used a multiethnic birth cohort at high-risk for most substance exposure. Future studies are needed to evaluate whether the methylation changes we identified are also present in other populations and biospecimens and determine whether the changes present at birth persist into later life stages. Additionally, despite the fact that this is perhaps among the largest DNA methylation studies of this kind, the sample size of individuals using three or more substances in our sample is small, and replication is needed. These findings have been partially replicated in the Environmental influences on Child Health Outcomes (ECHO) cohort by Schrott et al. 2025, however further work in high-risk subpopulations, such as those with a higher rate of mothers using three or more substances should be conducted to understand the nuances and limits of generalizability of these findings.

## Conclusions

We identified 72, 21, and 1 novel CpG showing suggestive methylation differences in cord blood at birth related to prenatal exposure to maternal use of opioids, cannabis, and polysubstance use during pregnancy, respectively, and built the first highly predictive methylation scores for opioids, cannabis, and polysubstance exposure during gestation. These results provide insight into neonate biology relevant to prenatal substance exposure, potential intervention targets, and an exposure classification tool for use in future studies.

## Supplementary Material

Epigenetics_2025Dec29_SupplementalFiles.docx

SupplFigureS7_Psub2.pdf

SuppFigureS5_Opioid.pdf

SupplFigureS6_Cannabis.pdf

SupplFiguresS8_Psub3.pdf

## Data Availability

The data underlying this article cannot be shared publicly due to the privacy of the individuals that participated in the study. The data will be shared at a reasonable request to the corresponding author after IRB review and approval. Summary statistics of our findings are available in this article and its supplementary material. Interested researchers may email BBCcontact@bmc.org with data and research requests, which are reviewed on a monthly basis. A preprint of a prior version of this paper is also publicly available [91].
