# Supplementary material for: Association of prenatal exposure to opioids, cannabis, and polysubstance use with cord blood DNA methylation patterns in a multiancestry cohort
Source: Epigenetics. 2026 Aug 26;21(1):2670885. doi: 10.1080/15592294.2026.2670885 (PMC13523970; doi:10.1080/15592294.2026.2670885)
Supplement: Supplementary file 1 [file KEPI_A_2670885_SM3932.pdf]

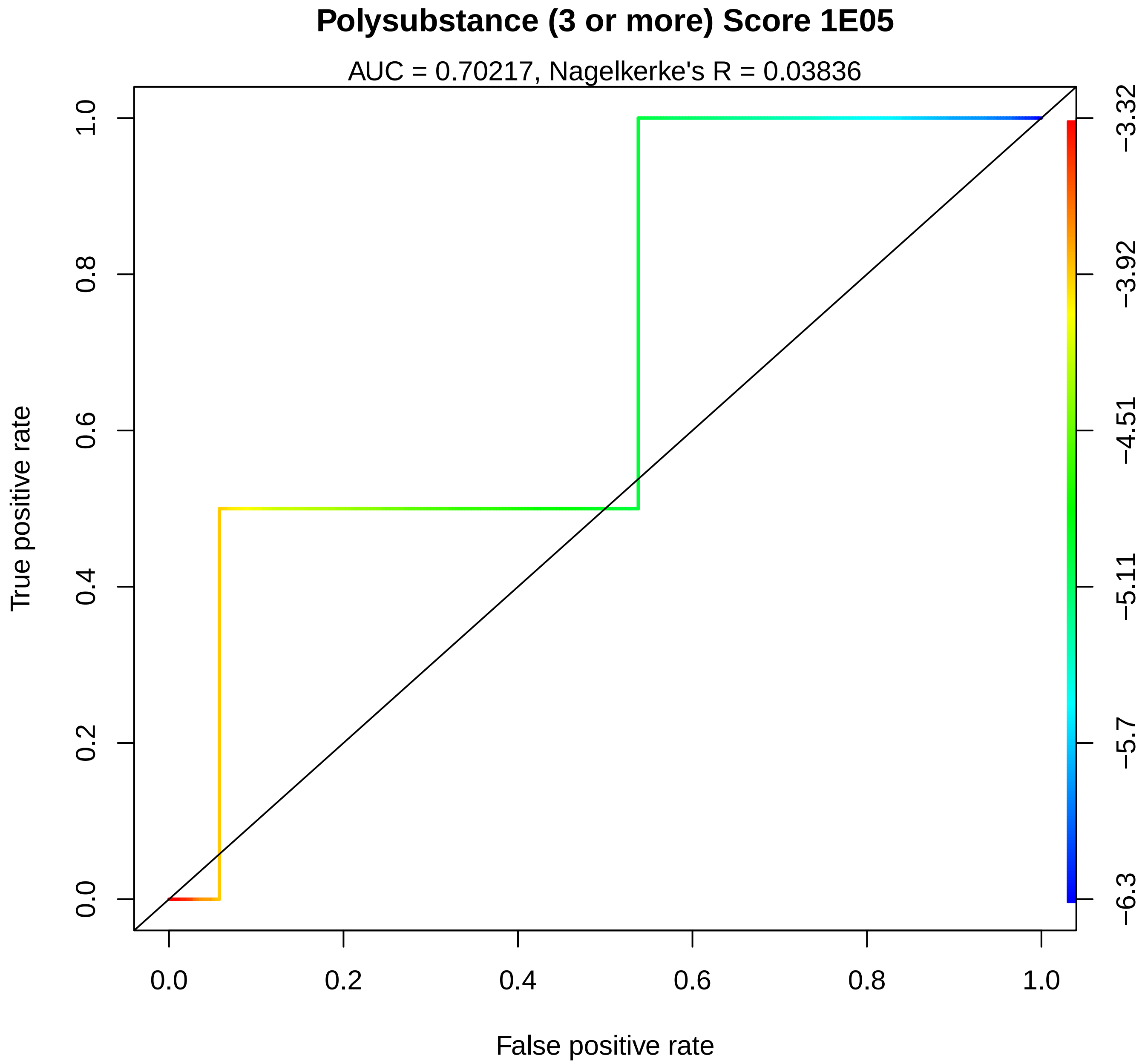

Polysubstance (3 or more) Score 1E05
AUC = 0.70217, Nagelkerke's R = 0.03836
True positive rate
False positive rate
0.0
0.2
0.4
0.6
0.8
1.0
−3.32
−3.92
−4.51
−5.11
−5.7
−6.3

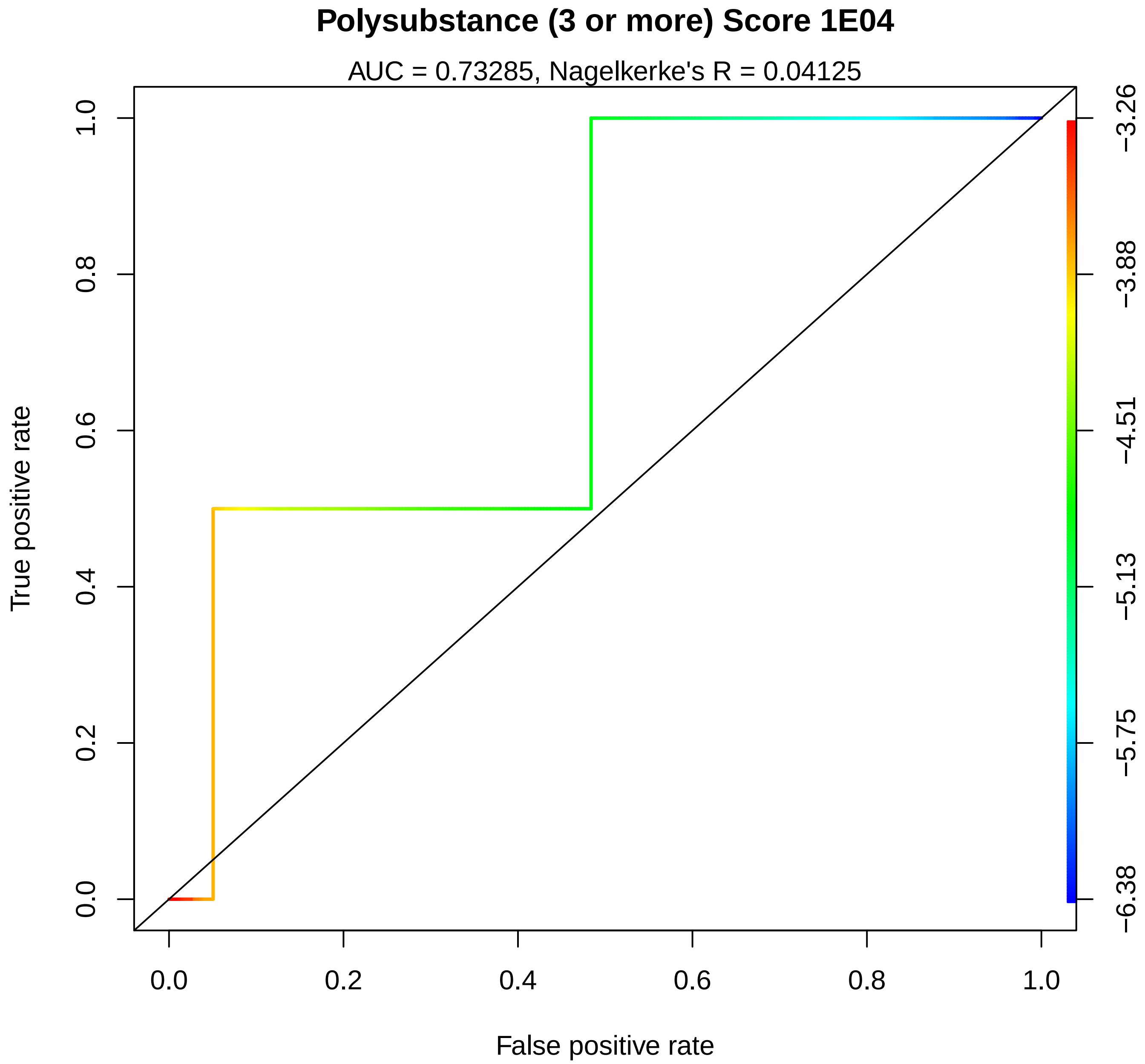

Polysubstance (3 or more) Score 1E04
AUC = 0.73285, Nagelkerke's R = 0.04125
True positive rate
False positive rate
0.0
0.2
0.4
0.6
0.8
1.0
−3.26
−3.88
−4.51
−5.13
−5.75
−6.38

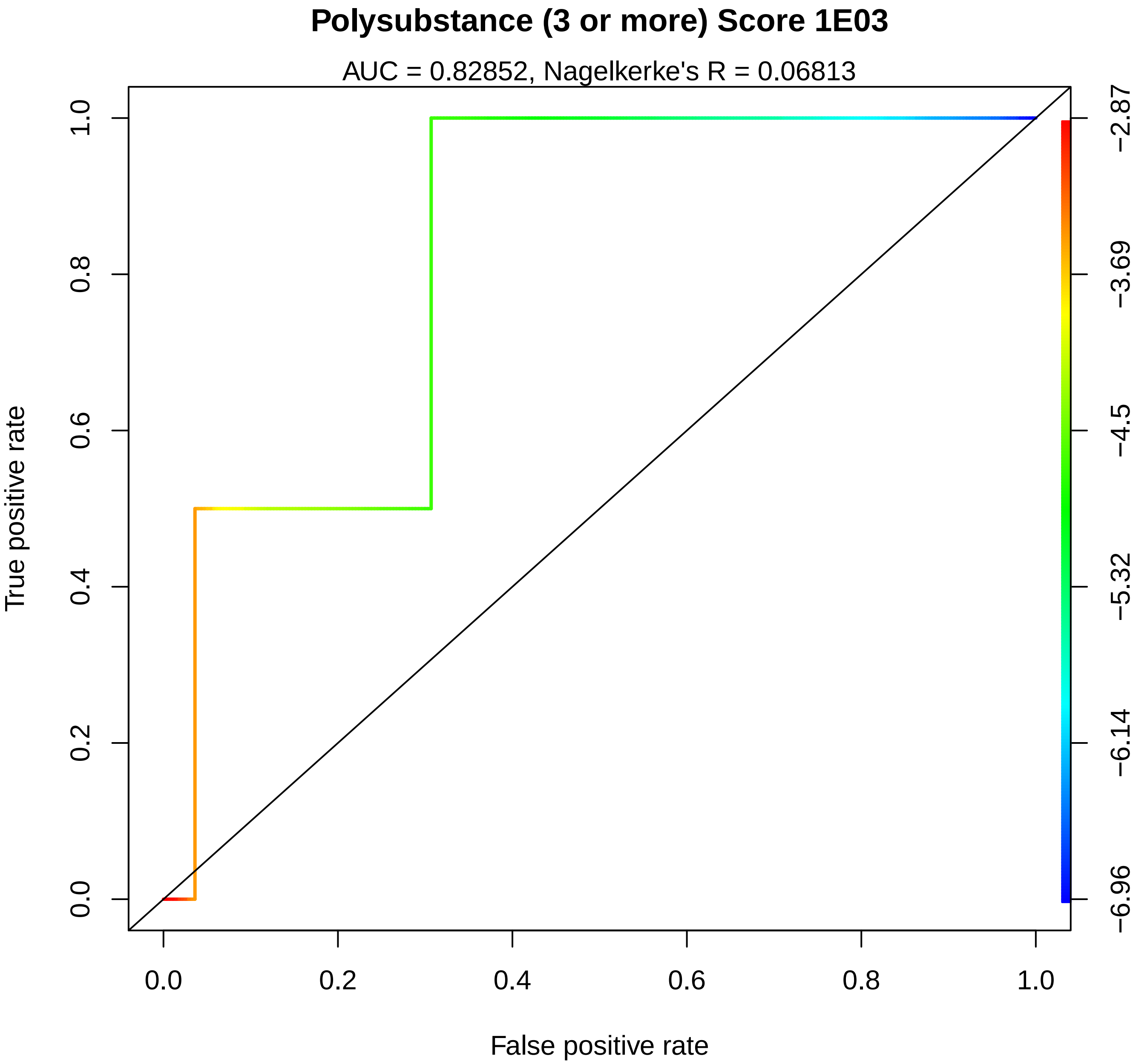
Polysubstance (3 or more) Score 1E03
AUC = 0.82852, Nagelkerke's R = 0.06813
True positive rate
False positive rate
0.0
0.2
0.4
0.6
0.8
1.0
−2.87
−3.69
−4.5
−5.32
−6.14
−6.96

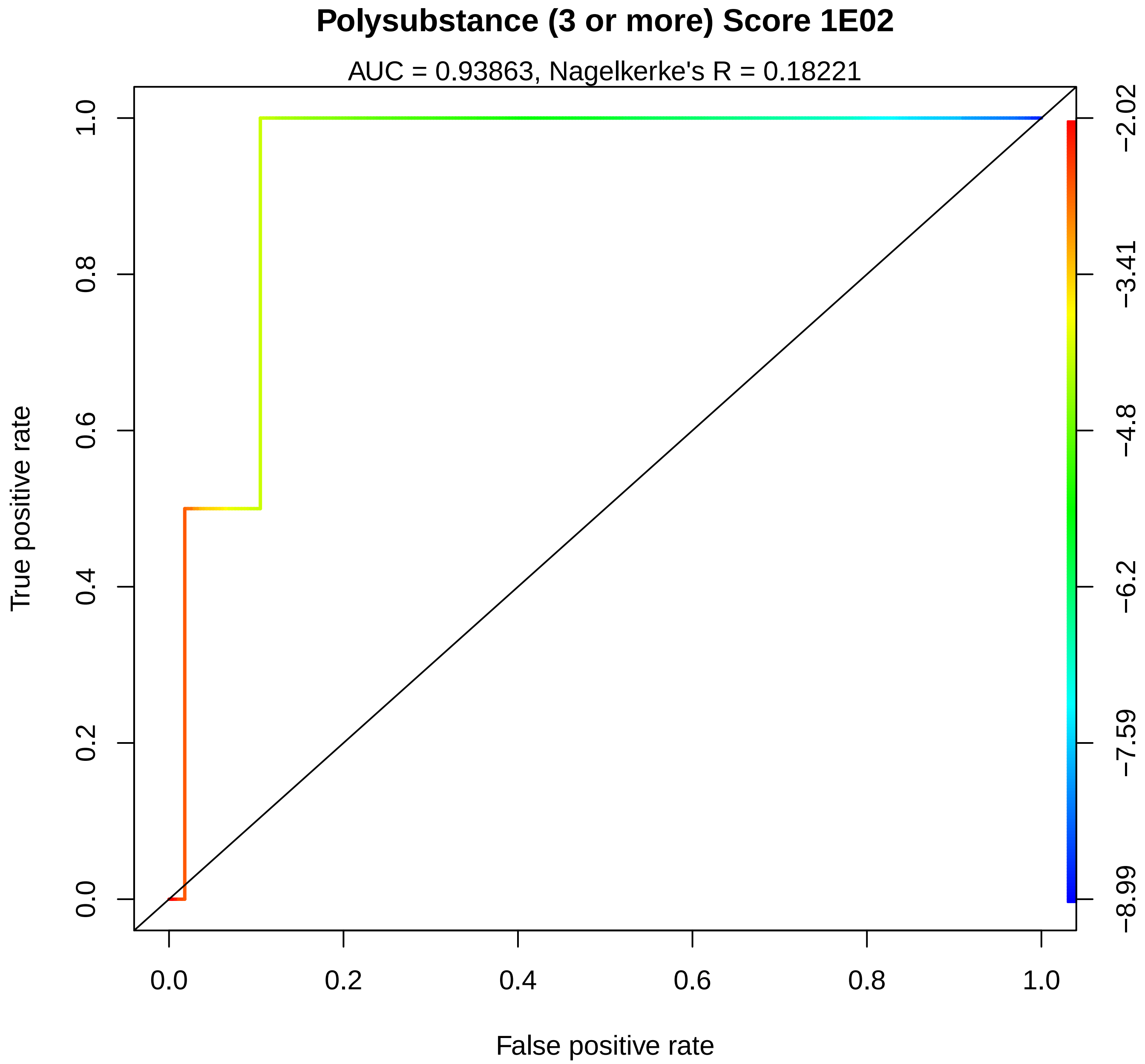
Polysubstance (3 or more) Score 1E02
AUC = 0.93863, Nagelkerke's R = 0.18221
True positive rate
False positive rate
0.0
0.2
0.4
0.6
0.8
1.0
−2.02
−3.41
−4.8
−6.2
−7.59
−8.99
