# Supplementary material for: Association of prenatal exposure to opioids, cannabis, and polysubstance use with cord blood DNA methylation patterns in a multiancestry cohort
Source: Epigenetics. 2026 Aug 26;21(1):2670885. doi: 10.1080/15592294.2026.2670885 (PMC13523970; doi:10.1080/15592294.2026.2670885)
Supplement: Supplementary file 2 [file KEPI_A_2670885_SM3933.pdf]

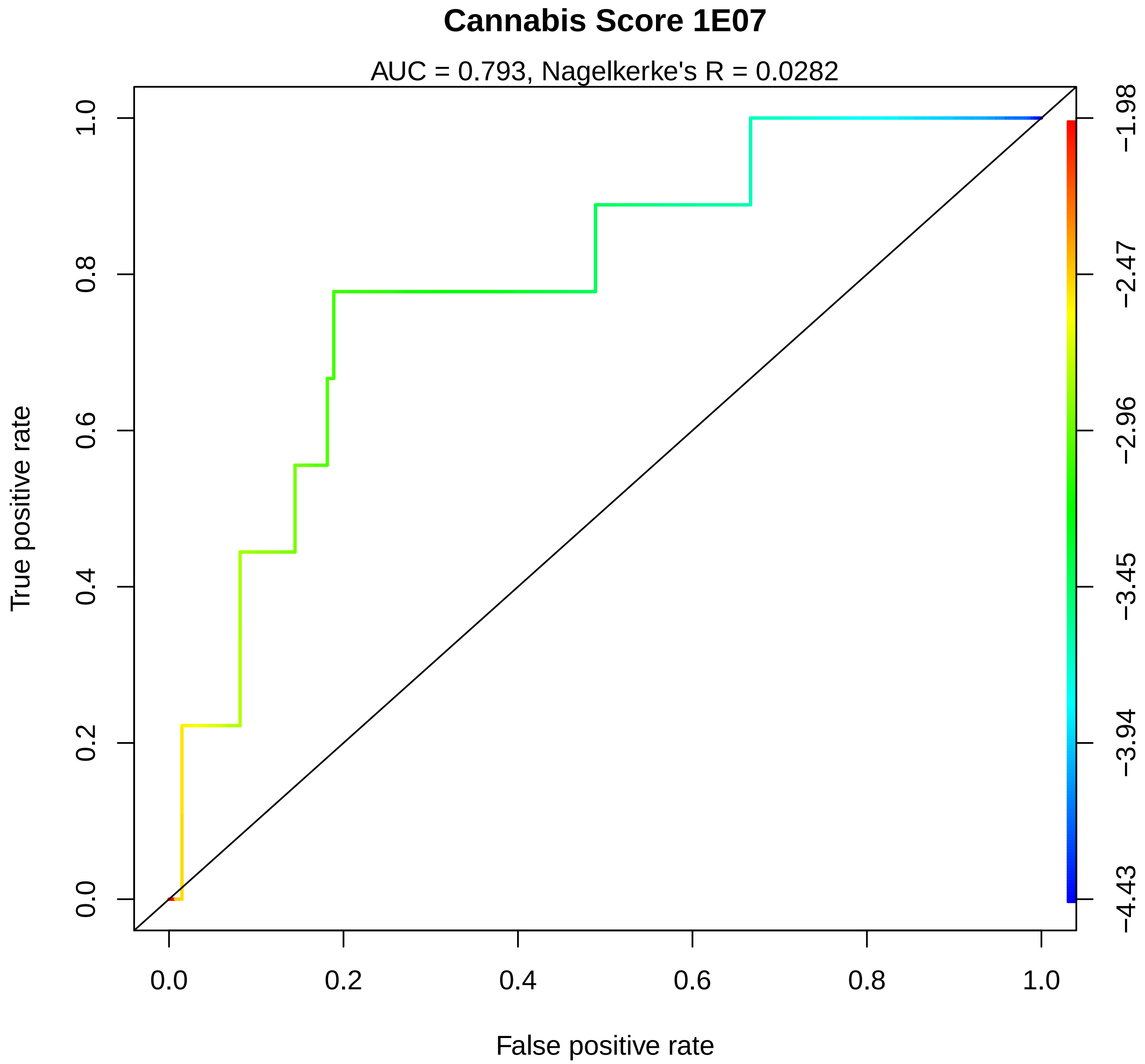
Cannabis Score 1E07
AUC = 0.793, Nagelkerke's R = 0.0282
True positive rate
False positive rate
0.0
0.2
0.4
0.6
0.8
1.0
−1.98
−2.47
−2.96
−3.45
−3.94
−4.43

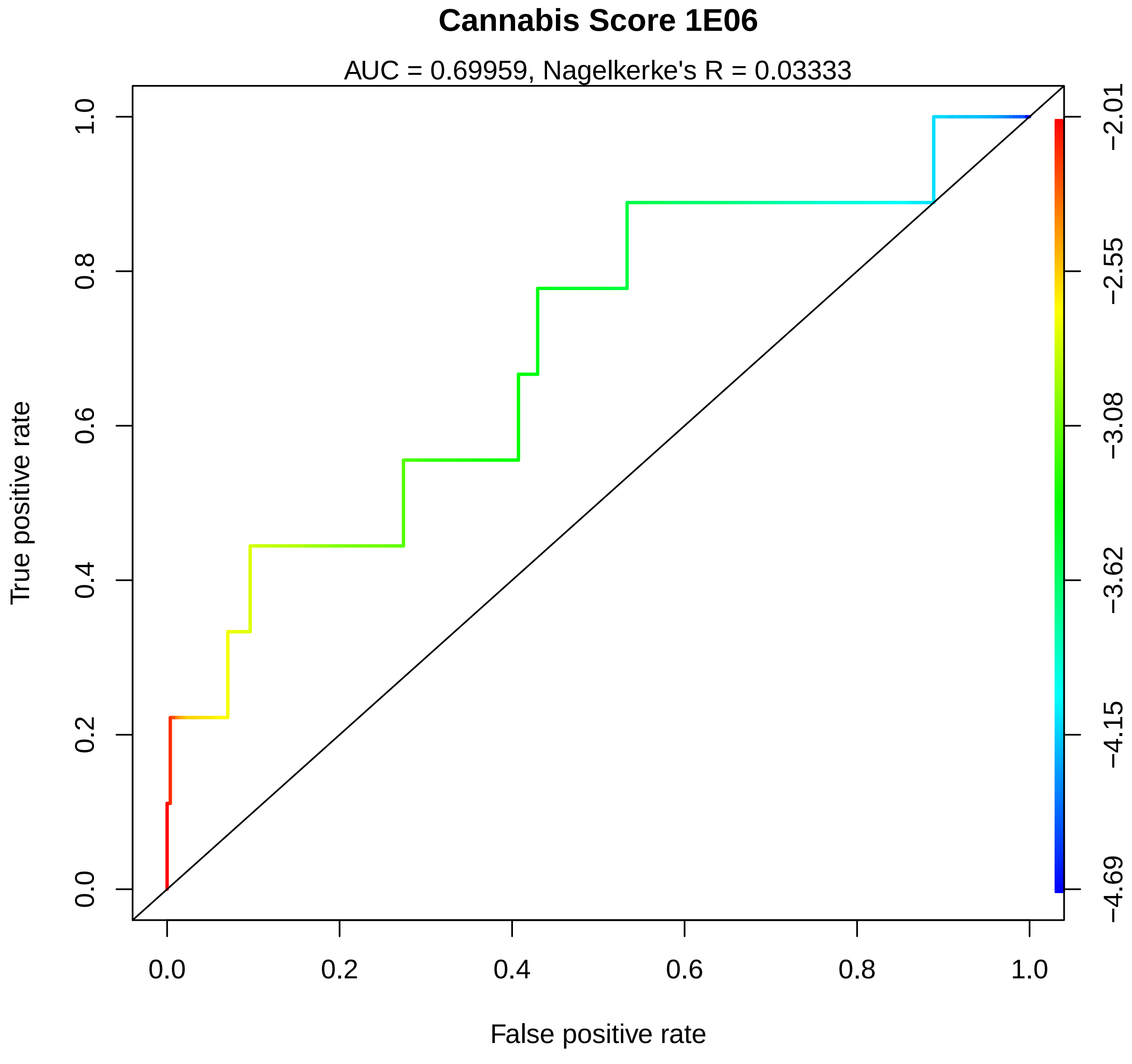
Cannabis Score 1E06
AUC = 0.69959, Nagelkerke's R = 0.03333
True positive rate
False positive rate
0.0
0.2
0.4
0.6
0.8
1.0
−2.01
−2.55
−3.08
−3.62
−4.15
−4.69

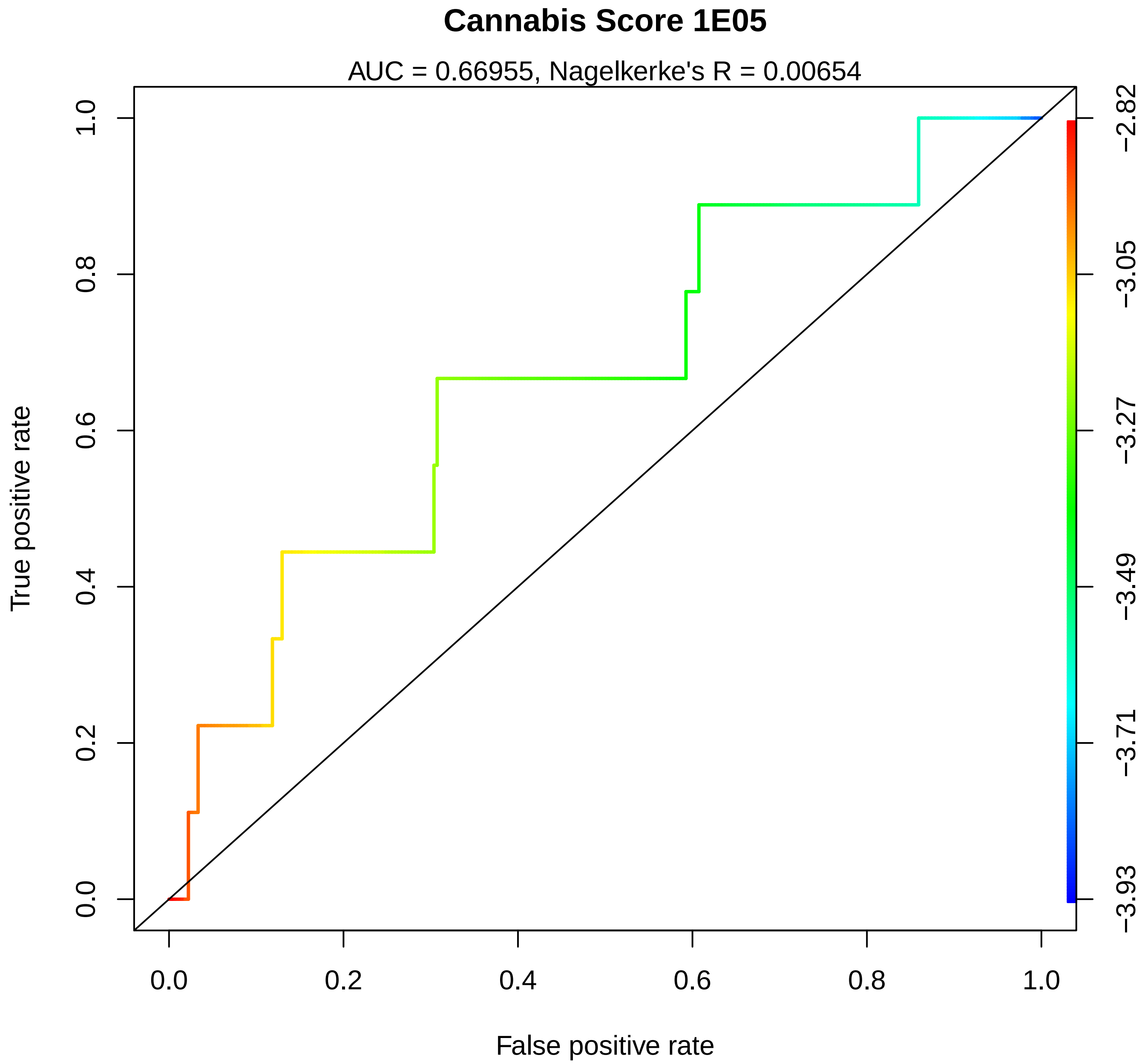

Cannabis Score 1E05
AUC = 0.66955, Nagelkerke's R = 0.00654
True positive rate
False positive rate
0.0
0.2
0.4
0.6
0.8
1.0
−2.82
−3.05
−3.27
−3.49
−3.71
−3.93

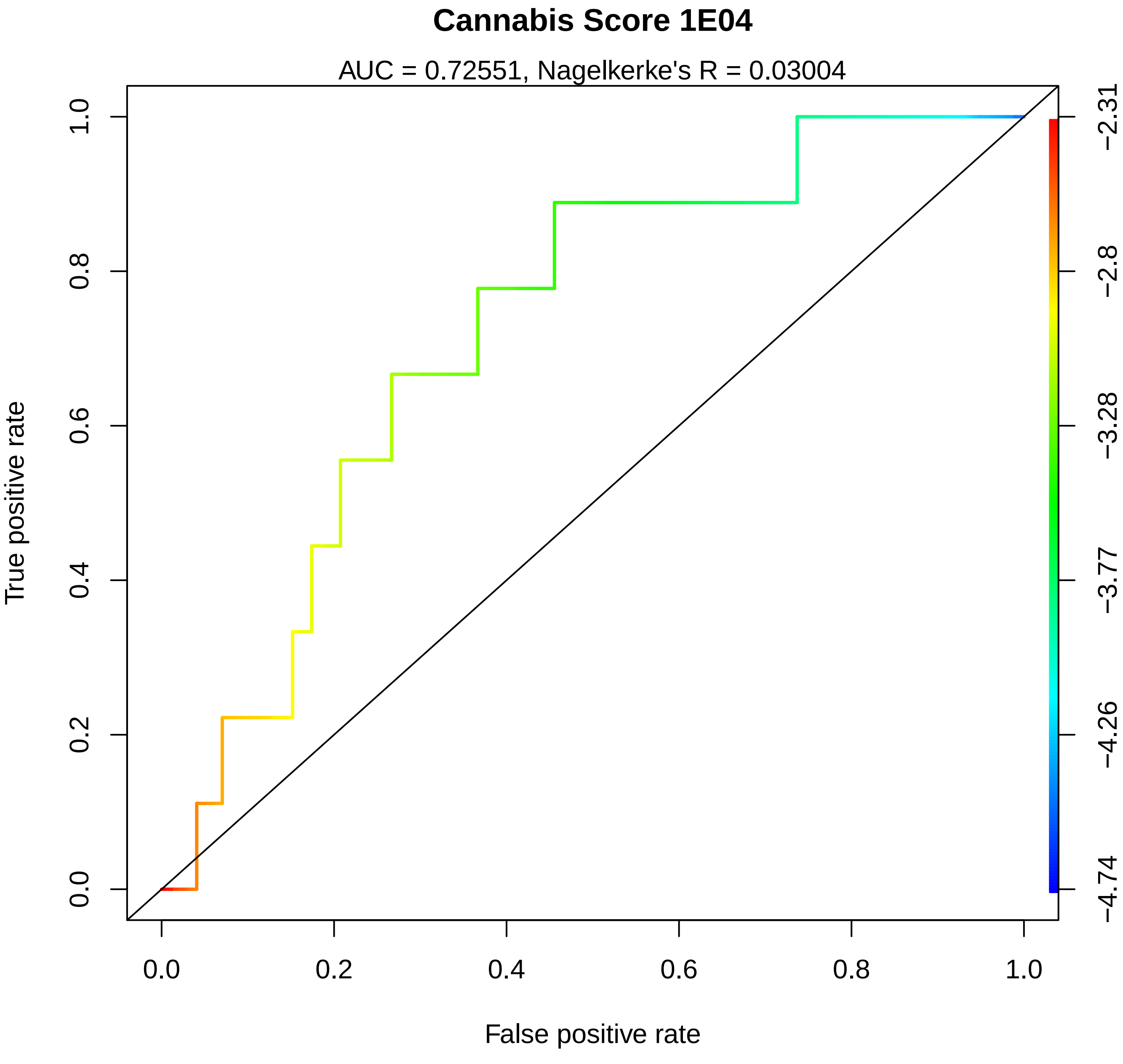

Cannabis Score 1E04
AUC = 0.72551, Nagelkerke's R = 0.03004
True positive rate
False positive rate
0.0
0.2
0.4
0.6
0.8
1.0
0.0
0.2
0.4
0.6
0.8
1.0
−2.31
−2.8
−3.28
−3.77
−4.26
−4.74

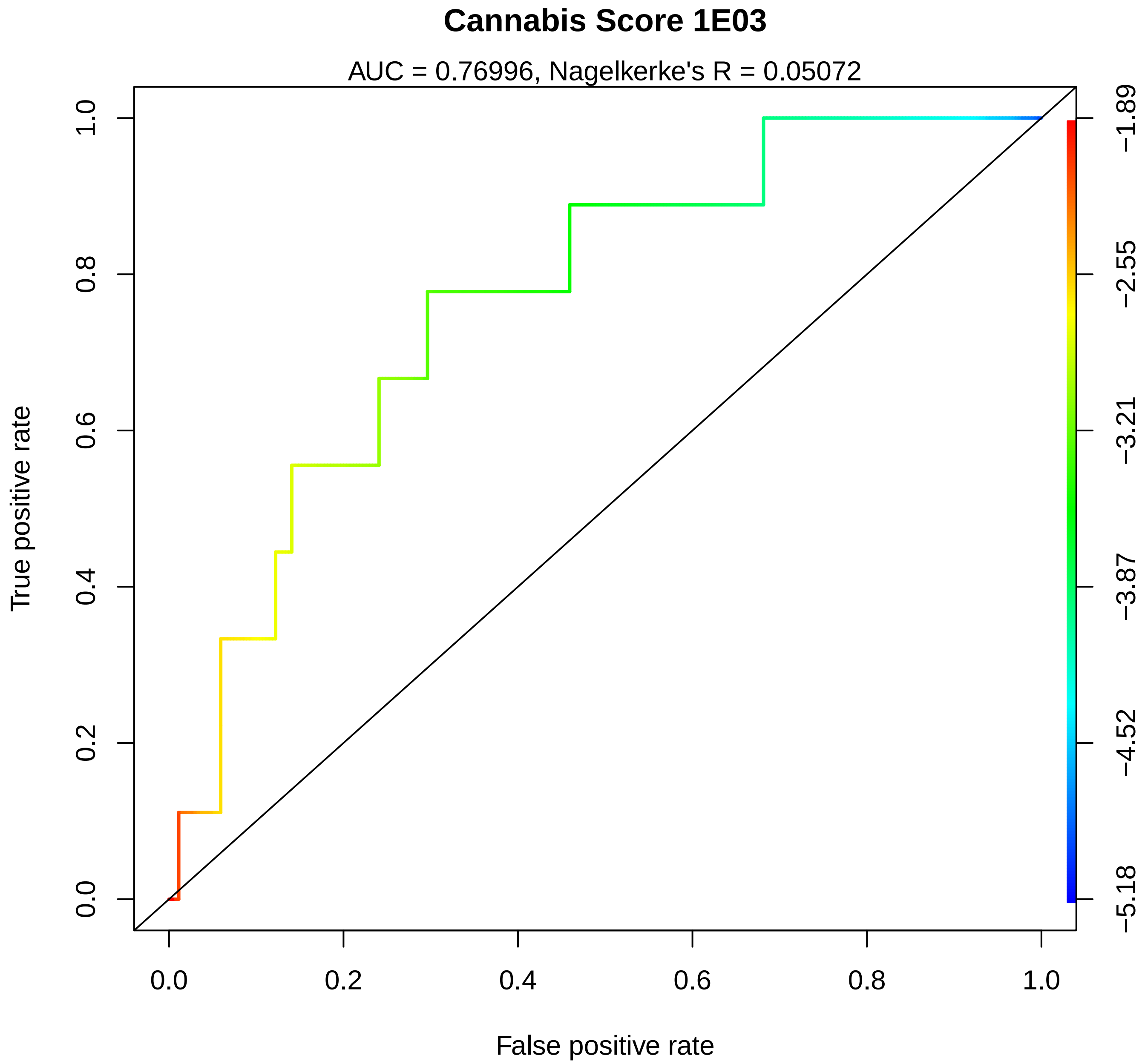

Cannabis Score 1E03
AUC = 0.76996, Nagelkerke's R = 0.05072
True positive rate
False positive rate
0.0
0.2
0.4
0.6
0.8
1.0
−1.89
−2.55
−3.21
−3.87
−4.52
−5.18

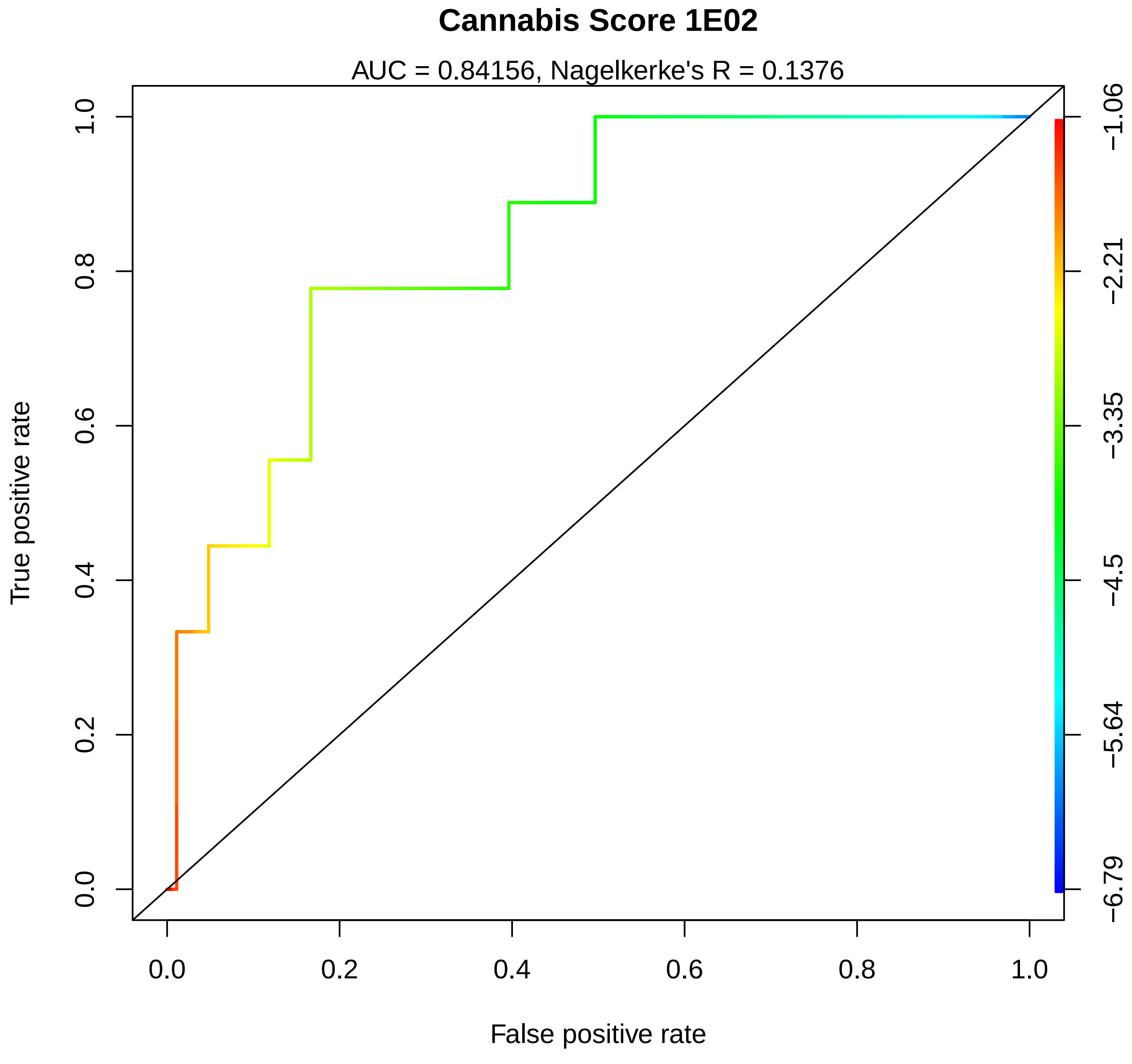
Cannabis Score 1E02
AUC = 0.84156, Nagelkerke's R = 0.1376
True positive rate
False positive rate
0.0
0.2
0.4
0.6
0.8
1.0
−1.06
−2.21
−3.35
−4.5
−5.64
−6.79
