# Supplementary material for: Association of prenatal exposure to opioids, cannabis, and polysubstance use with cord blood DNA methylation patterns in a multiancestry cohort
Source: Epigenetics. 2026 Aug 26;21(1):2670885. doi: 10.1080/15592294.2026.2670885 (PMC13523970; doi:10.1080/15592294.2026.2670885)
Supplement: Supplementary file 3 [file KEPI_A_2670885_SM3934.pdf]

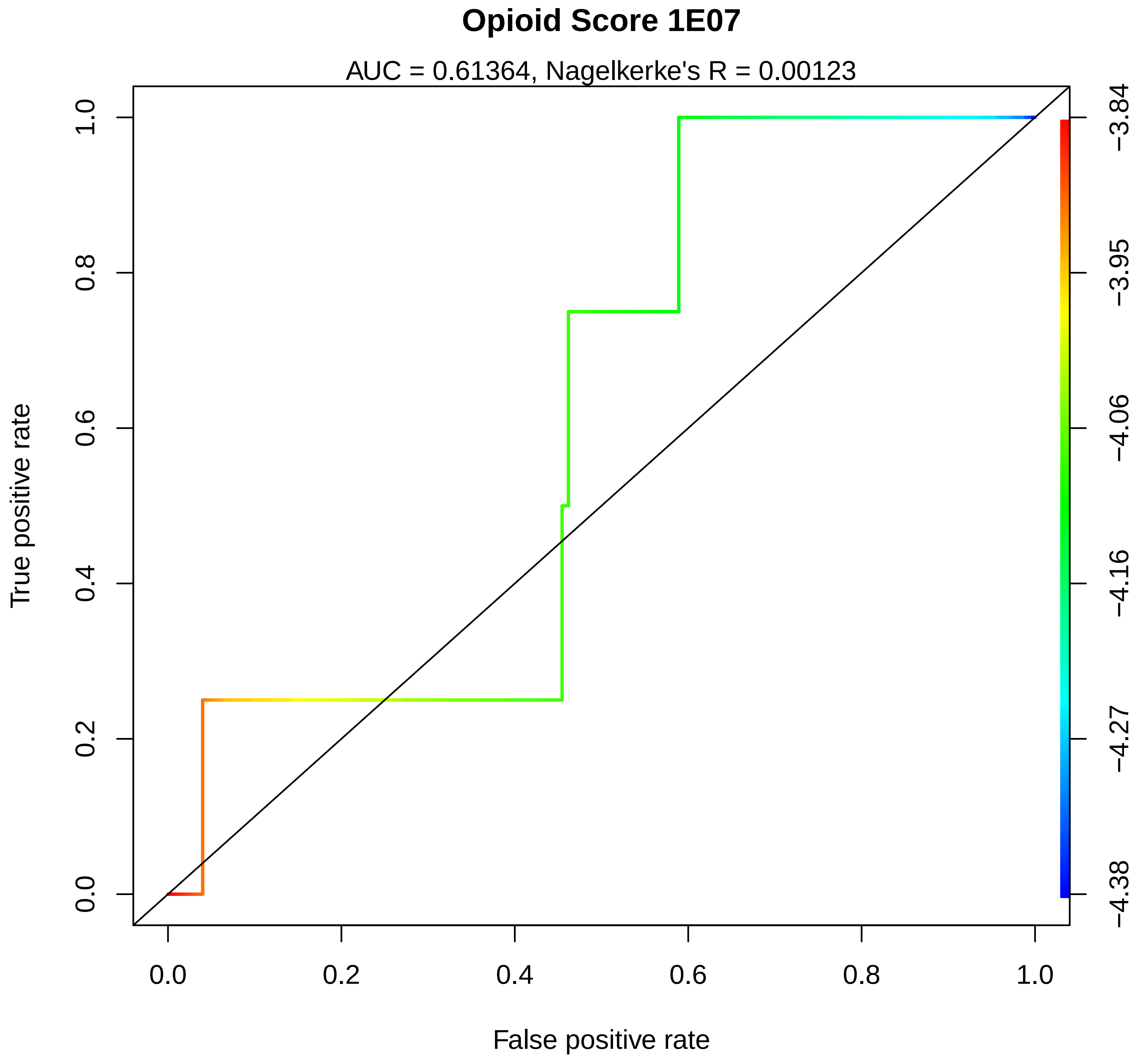

Opioid Score 1E07
AUC = 0.61364, Nagelkerke's R = 0.00123
True positive rate
False positive rate
0.0
0.2
0.4
0.6
0.8
1.0
−3.84
−3.95
−4.06
−4.16
−4.27
−4.38

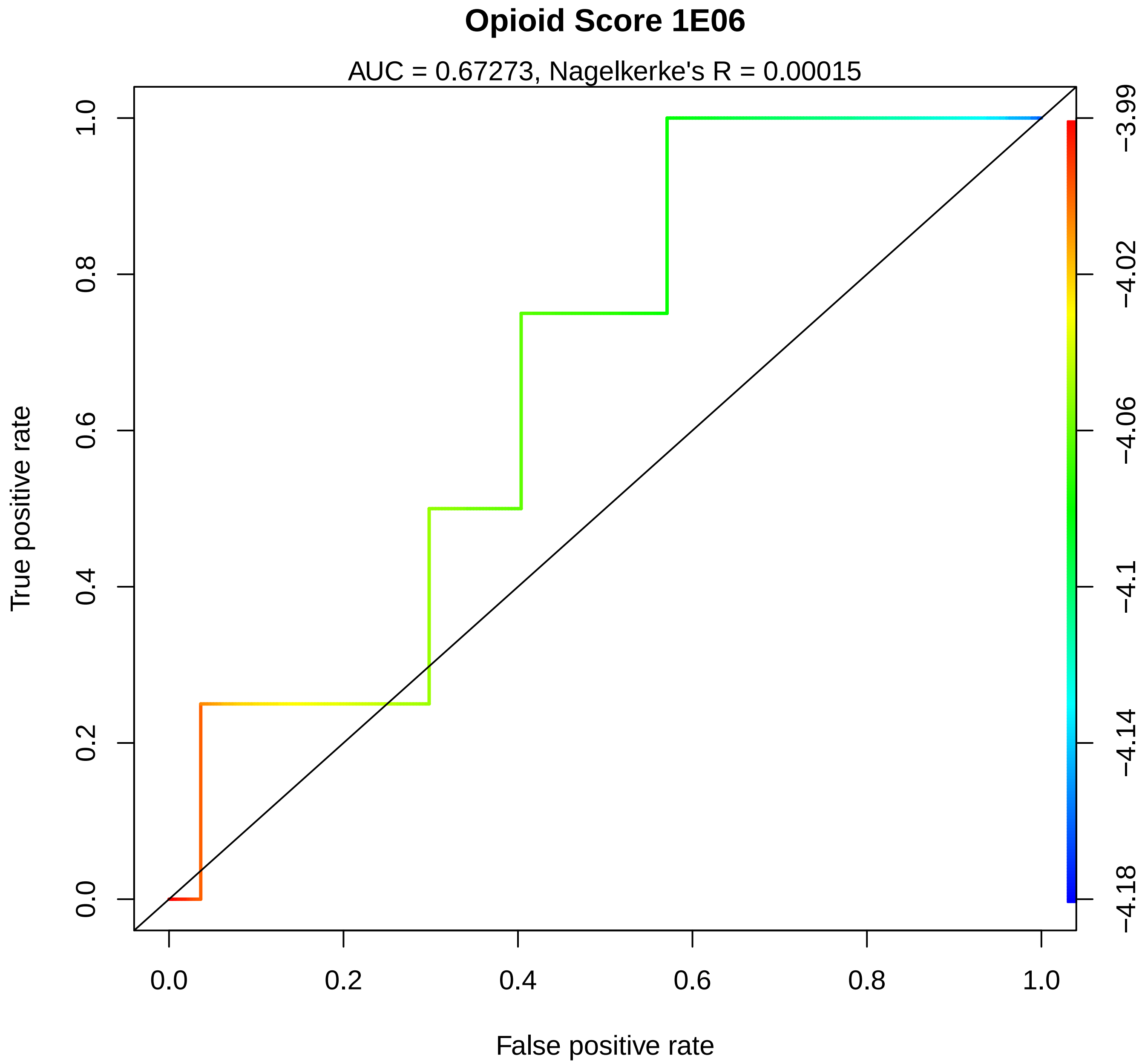

Opioid Score 1E06
AUC = 0.67273, Nagelkerke's R = 0.00015
True positive rate
False positive rate
0.0
0.2
0.4
0.6
0.8
1.0
−3.99
−4.02
−4.06
−4.1
−4.14
−4.18

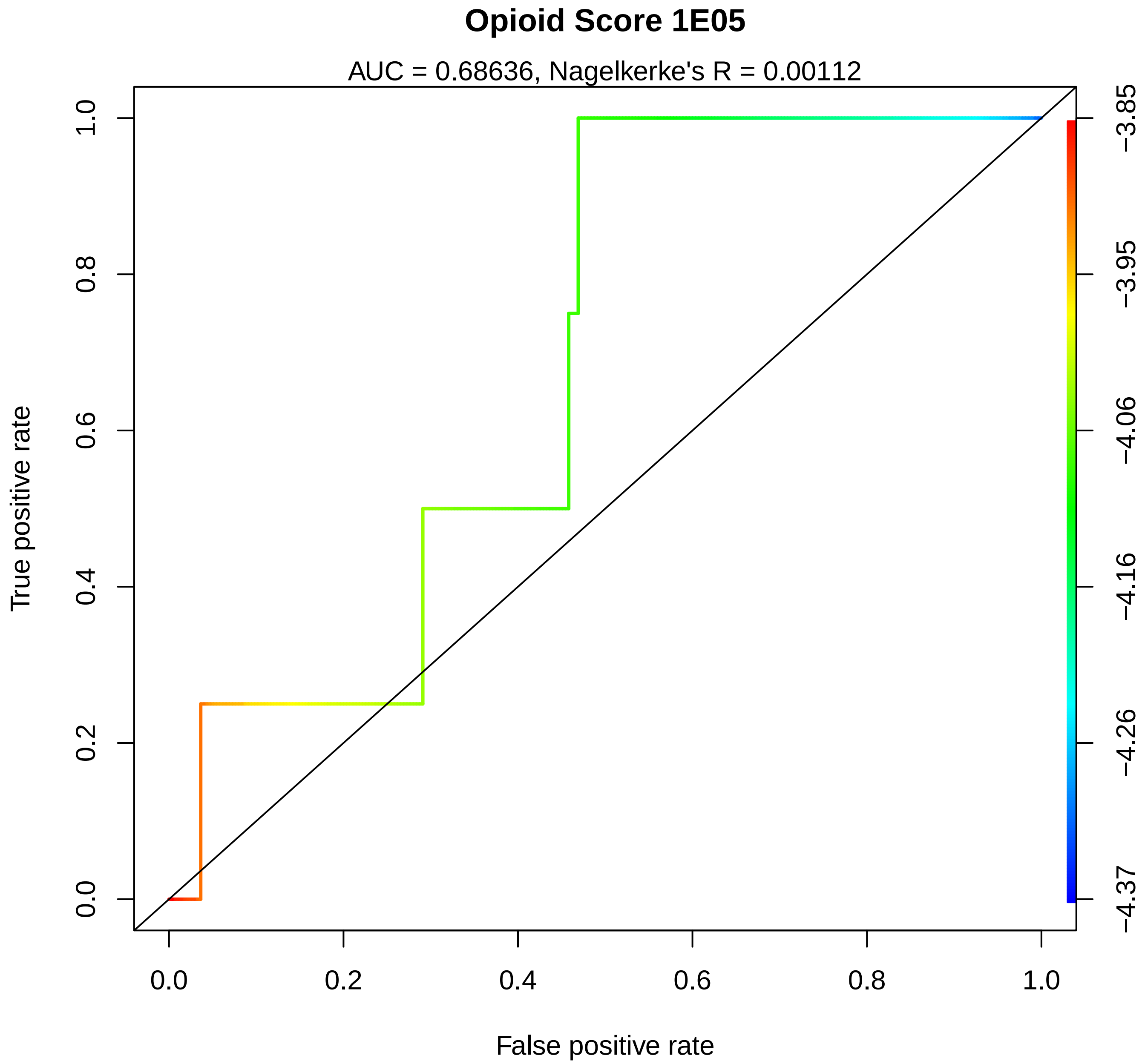
Opioid Score 1E05
AUC = 0.68636, Nagelkerke's R = 0.00112
True positive rate
False positive rate
0.0
0.2
0.4
0.6
0.8
1.0
−3.85
−3.95
−4.06
−4.16
−4.26
−4.37

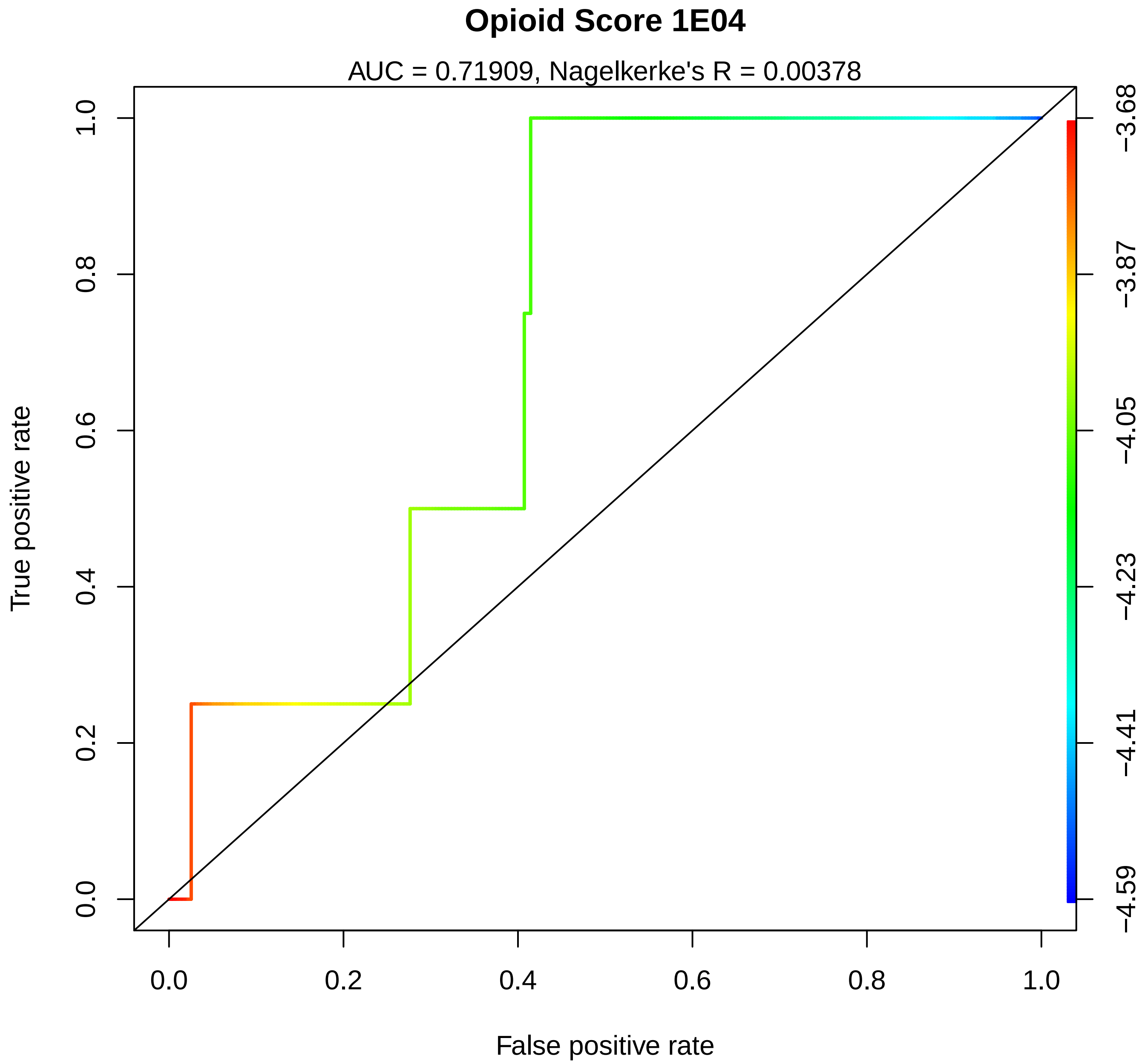

Opioid Score 1E04
AUC = 0.71909, Nagelkerke's R = 0.00378
True positive rate
False positive rate
0.0
0.2
0.4
0.6
0.8
1.0
−3.68
−3.87
−4.05
−4.23
−4.41
−4.59

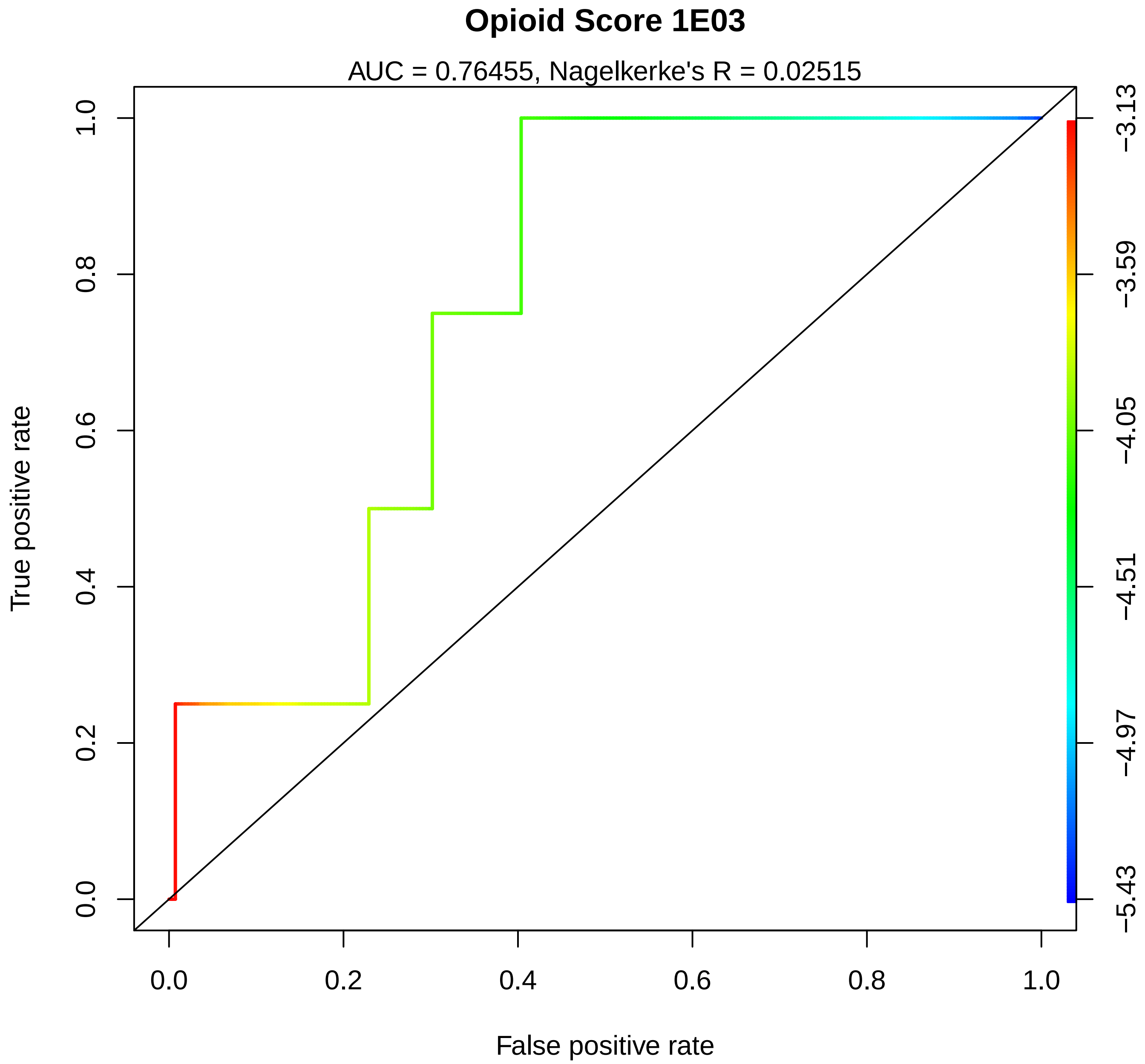

Opioid Score 1E03
AUC = 0.76455, Nagelkerke's R = 0.02515
True positive rate
False positive rate
0.0
0.2
0.4
0.6
0.8
1.0
−3.13
−3.59
−4.05
−4.51
−4.97
−5.43

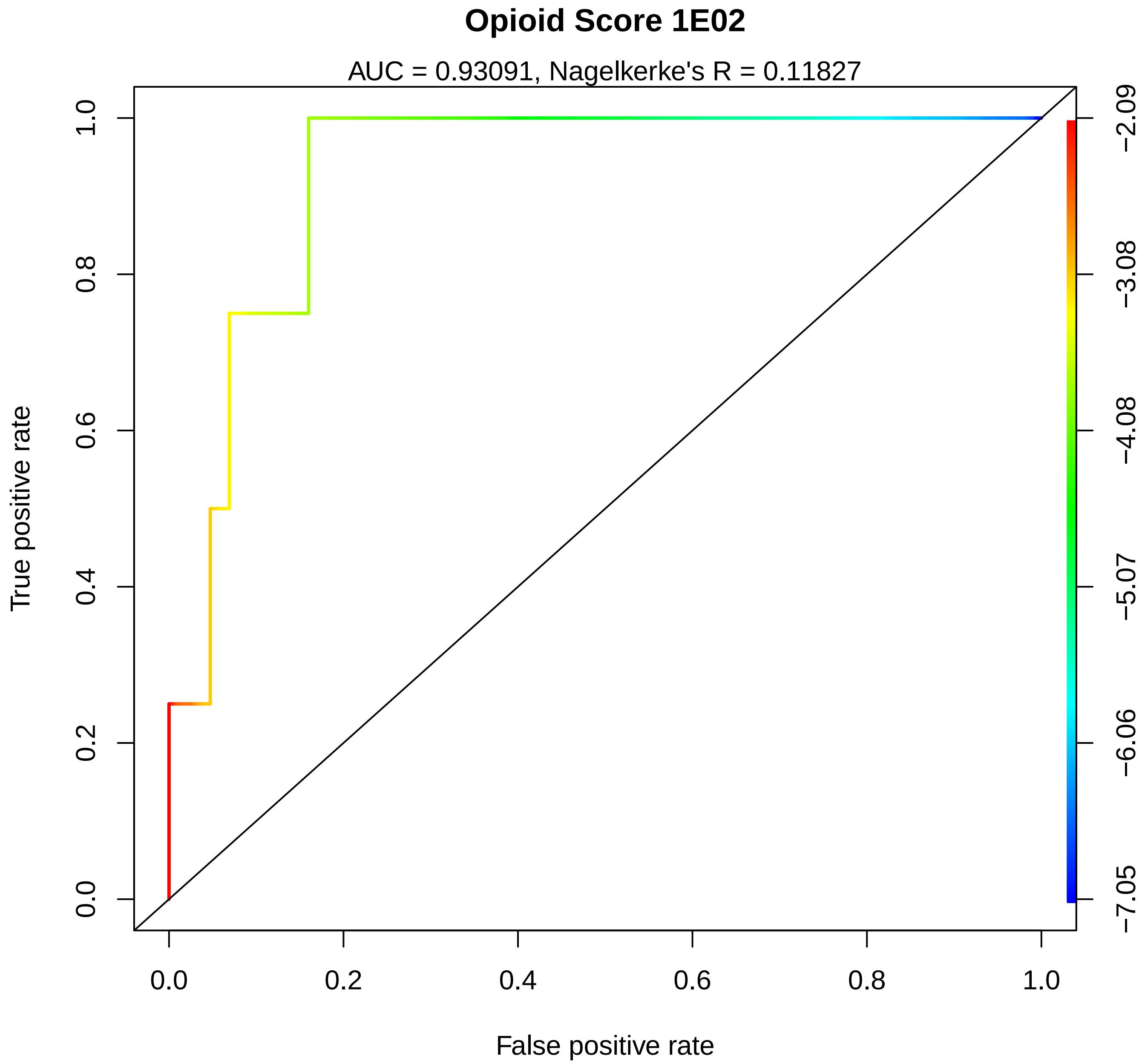
Opioid Score 1E02
AUC = 0.93091, Nagelkerke's R = 0.11827
True positive rate
False positive rate
0.0
0.2
0.4
0.6
0.8
1.0
−2.09
−3.08
−4.08
−5.07
−6.06
−7.05
