# Supplementary material for: Association of prenatal exposure to opioids, cannabis, and polysubstance use with cord blood DNA methylation patterns in a multiancestry cohort
Source: Epigenetics. 2026 Aug 26;21(1):2670885. doi: 10.1080/15592294.2026.2670885 (PMC13523970; doi:10.1080/15592294.2026.2670885)
Supplement: Supplementary file 4 [file KEPI_A_2670885_SM3935.pdf]

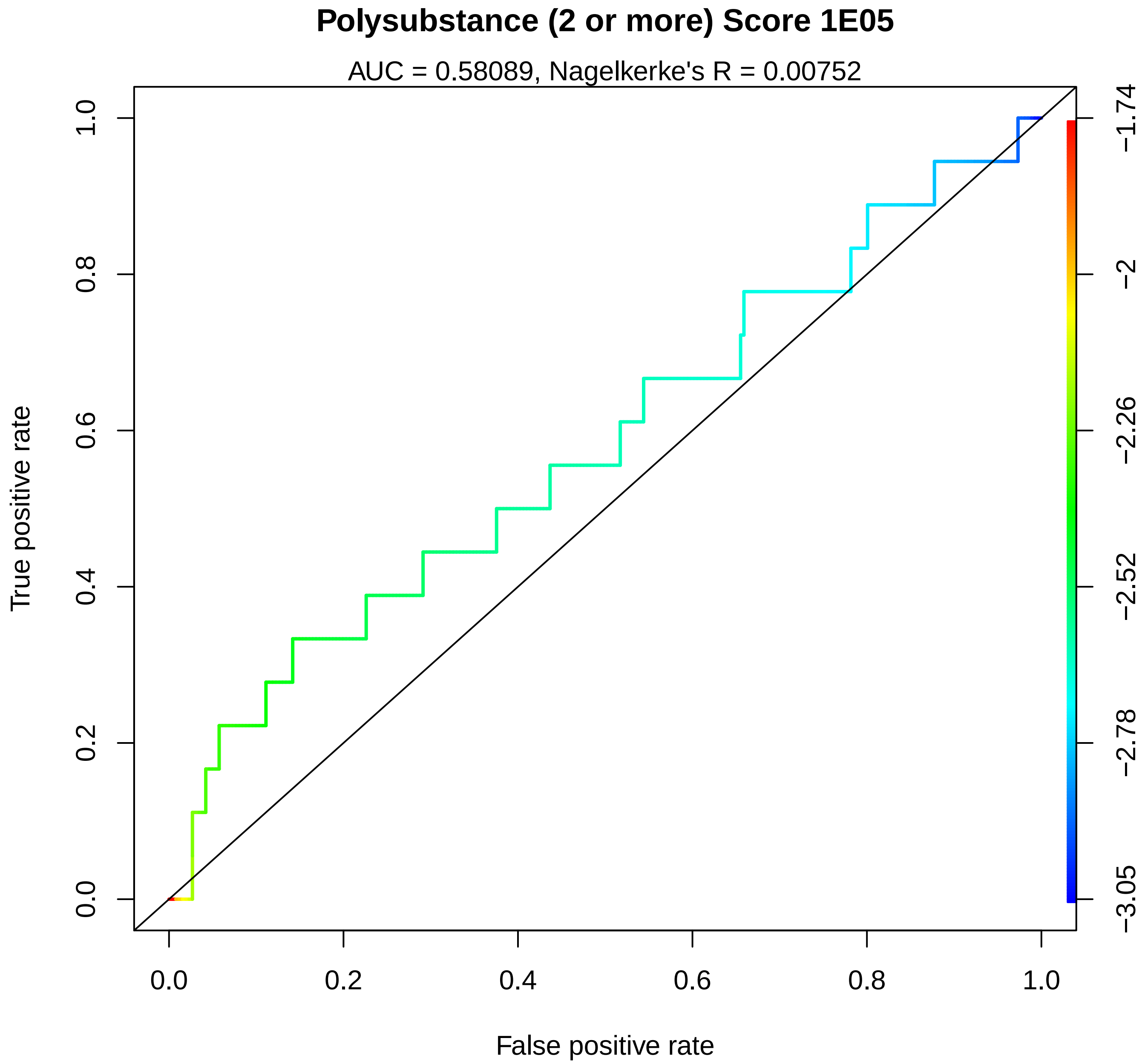

Polysubstance (2 or more) Score 1E05
AUC = 0.58089, Nagelkerke's R = 0.00752
True positive rate
False positive rate
0.0
0.2
0.4
0.6
0.8
1.0
−1.74
−2
−2.26
−2.52
−2.78
−3.05

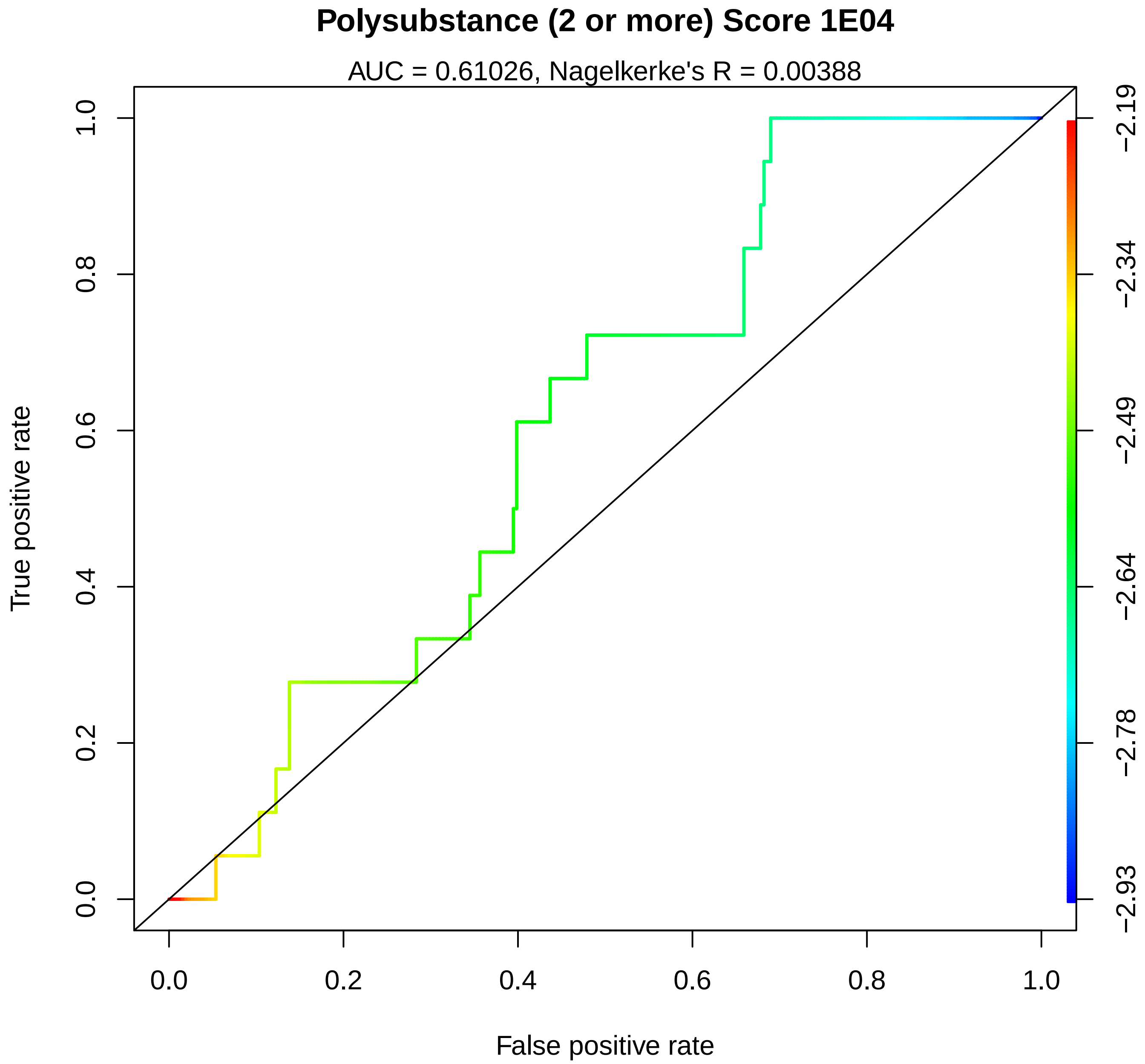
Polysubstance (2 or more) Score 1E04
AUC = 0.61026, Nagelkerke's R = 0.00388
True positive rate
False positive rate
0.0
0.2
0.4
0.6
0.8
1.0
−2.19
−2.34
−2.49
−2.64
−2.78
−2.93

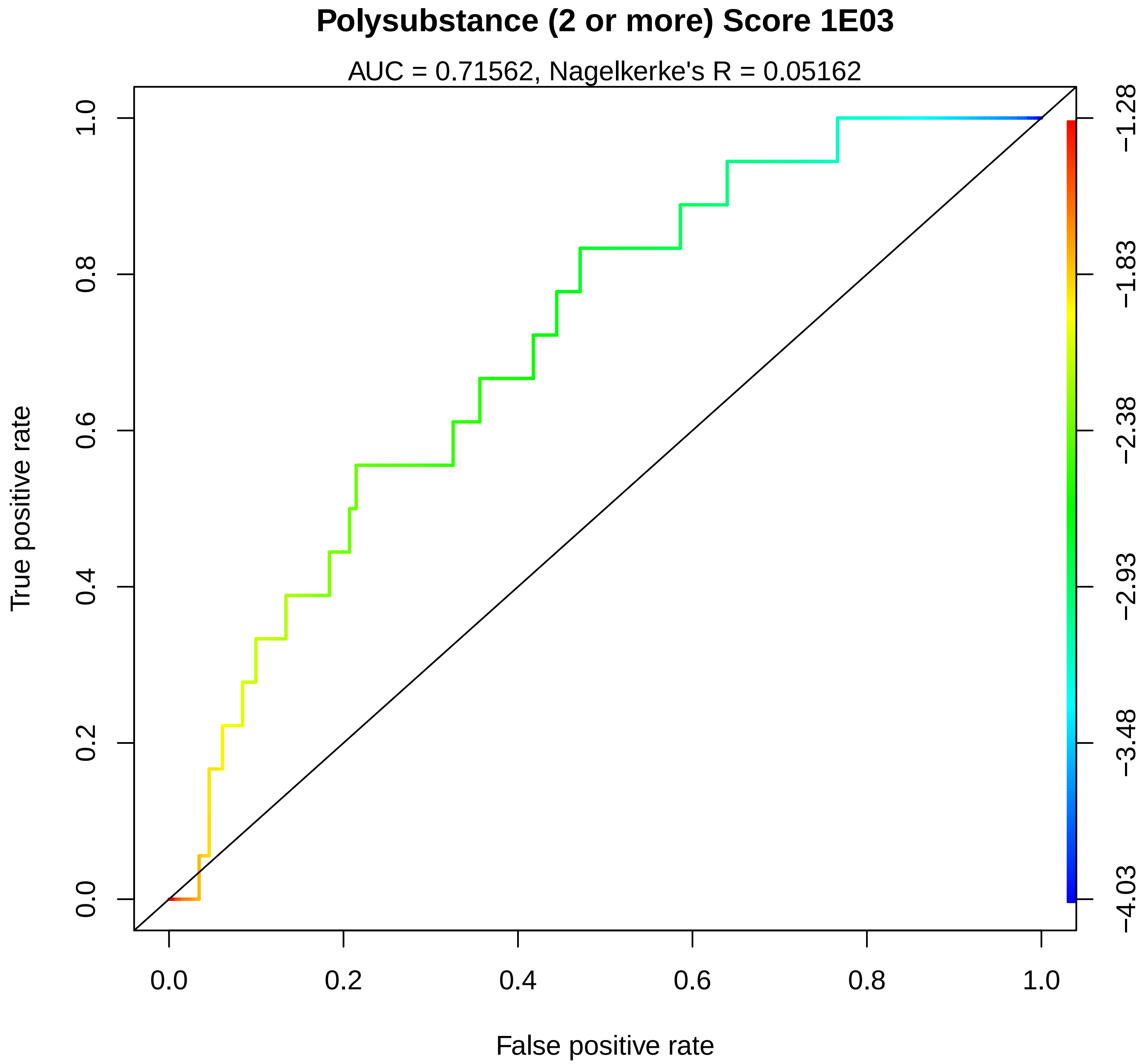

Polysubstance (2 or more) Score 1E03
AUC = 0.71562, Nagelkerke's R = 0.05162
True positive rate
False positive rate
0.0
0.2
0.4
0.6
0.8
1.0
−1.28
−1.83
−2.38
−2.93
−3.48
−4.03

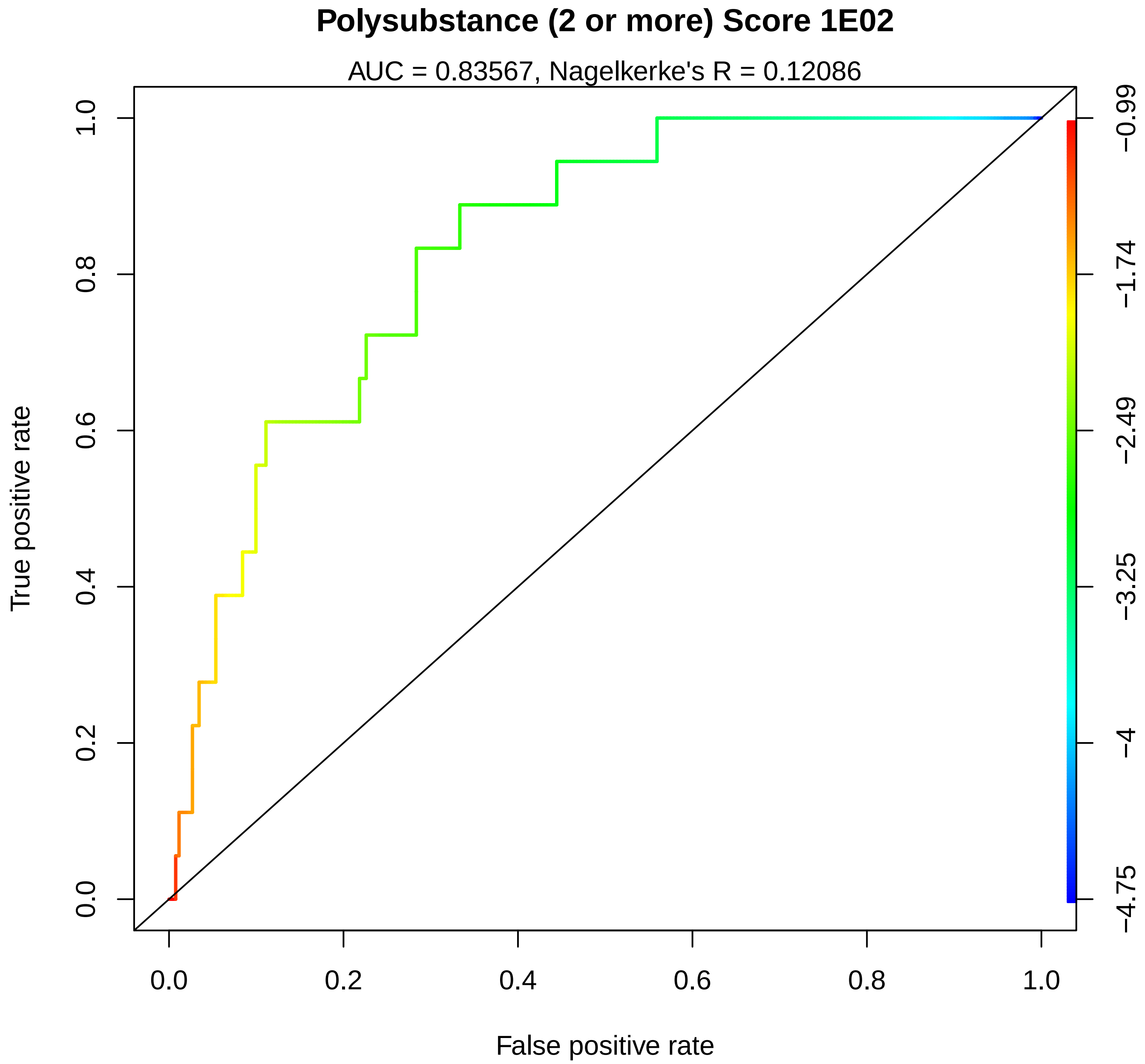

Polysubstance (2 or more) Score 1E02
AUC = 0.83567, Nagelkerke's R = 0.12086
True positive rate
False positive rate
0.0
0.2
0.4
0.6
0.8
1.0
−0.99
−1.74
−2.49
−3.25
−4
−4.75
