# Supplementary material for: Association of prenatal exposure to opioids, cannabis, and polysubstance use with cord blood DNA methylation patterns in a multiancestry cohort
Source: Epigenetics. 2026 Aug 26;21(1):2670885. doi: 10.1080/15592294.2026.2670885 (PMC13523970; doi:10.1080/15592294.2026.2670885)
Supplement: Supplementary file 5 [file KEPI_A_2670885_SM3936.docx]

## Supplementary Material.

Supplementary Figure S1. Flowchart of study population inclusion from baseline cohort to analytic sample.

.

Supplementary Figure S2. Flowchart of epigenetic data for probe-based and sample-based quality control criteria.

Supplementary Figure S3. Conceptual diagram of data partition and testing for epigenetic score cutoff assessment and final score construction.

Supplementary Figure S4. Quantile-quantile plot of p-values for epigenome-wide association analyses and opioid, cannabis, and polysubstance exposures in the Boston Birth Cohort.

**Supplementary Table S1.** Distribution of specific substance use among polysubstance exposed samples.

| Polysubstance (2+) N=65 | Alcohol | Cannabis | Opioids |
| --- | --- | --- | --- |
| Tobacco | 43 (66.2%) | 26 (40.0%) | 13 (20.0%) |
| Alcohol | X | 8 (12.3%) | 3 (4.6%) |
| Cannabis | X | X | 1 (1.5%) |

Supplementary Table S2. Epigenetic loci with significant or suggestive associations with opioid exposure from epigenome-wide analysis in the Boston Birth Cohort.

<https://drive.google.com/file/d/1esusUuMUvjg35J0iFAFR64irZHucdbNc/view?usp=drive_link>

Supplementary Table S3 . Epigenetic loci with significant or suggestive associations with cannabis exposure from epigenome-wide analysis in the Boston Birth Cohort.

<https://drive.google.com/file/d/1wWPpK2VIvPFWxo_mgTFID7X8ao9Aysia/view?usp=drive_link>

Supplementary Table S4. Epigenetic loci with significant or suggestive associations with polysubstance exposure, defined as 2 or more substances, from epigenome-wide analysis in the Boston Birth Cohort.

<https://drive.google.com/file/d/1coLJMRqVo2Tt4tx4rHkYDogGMqUquAlY/view?usp=drive_link>

Supplementary Table S5. Epigenetic loci with significant or suggestive associations with polysubstance exposure, defined as 3 or more substances, from epigenome-wide analysis in the Boston Birth Cohort.

<https://drive.google.com/file/d/1lMv3oyLcxMkhArC__EDrOAS7zNPRDjWR/view?usp=drive_link>

**Supplemental Figure S5. Receiver operating characteristic (ROC) curves of opioid polyepigenetic scores for test datasets calculated to determine the best p-value cutoff.**

*See attached supplementary figure file.*

**Supplemental Figure S6. Receiver operating characteristic (ROC) curves of cannabis polyepigenetic scores for test datasets calculated to determine the best p-value cutoff.**

*See attached supplementary figure file.*

**Supplemental Figure S7. Receiver operating characteristic (ROC) curves of polysubstance (2+ substances) polyepigenetic scores for test datasets calculated to determine the best p-value cutoff.**

*See attached supplementary figure file.*

**Supplemental Figure S8. Receiver operating characteristic (ROC) curves of polysubstance (3+ substances) polyepigenetic scores for test datasets calculated to determine the best p-value cutoff.**

*See attached supplementary figure file.*

**Supplementary Table S6. Associations between opioid and cannabis polyepigenetic score and all substance exposures using logistic regression and controlling for socioedemographic confounders.**

|  | Opioids  OR (95% CI) | Cannabis  OR (95% CI) | Tobacco  OR (95% CI) | Alcohol  OR (95% CI) |
| --- | --- | --- | --- | --- |
| Scaled opioid score | 2.29 (1.27, 4.31) | 0.87 (0.58, 1.30) | 1.14 (0.96, 1.36) | 1.11 (0.86, 1.44) |
| Scaled cannabis score | 0.75 (0.43, 1.29) | 2.10 (1.39, 3.24) | 1.15 (0.94, 1.41) | 1.16 (0.90, 1.50) |

OR: Odds ratio; 95% CI: 95% Confidence Interval

Controlling for maternal age, child’s sex, pre-pregnancy maternal body-mass-index, maternal education, maternal race/ethnicity, household yearly income, and child’s gestational age at birth.

**Supplementary Table S7.** **Spearman rank correlation of overlapping loci from epigenetic scores.**

|  | Cannabis score | Opioid Score | PACE tobacco loci |
| --- | --- | --- | --- |
| Opioid score | 0.783 |  |  |
| PACE tobacco loci | 0.400 | 0.621 |  |
| PACE alcohol loci | 0.500 | 0.119 | -0.05 |

Correlation coefficients were calculated only among loci that were included in each respective score/EWAS. For examine, the 0.783 coefficient only reflects 7 loci that were included in both the cannabis polyepigenetic score and the opioid epigenetic score with p-value threshold <1e-03. Loci that were only included in a single score (for instance, only present in the opioid score and not in the cannabis score) were excluded from analysis, as the correlation would automatically be 0.
